# Shifts in microbial diversity, composition, and functionality in the gut and genital microbiome during a natural SIV infection in vervet monkeys

**DOI:** 10.1186/s40168-020-00928-4

**Published:** 2020-11-06

**Authors:** Anna J. Jasinska, Tien S. Dong, Venu Lagishetty, William Katzka, Jonathan P. Jacobs, Christopher A. Schmitt, Jennifer Danzy Cramer, Dongzhu Ma, Willem G. Coetzer, J. Paul Grobler, Trudy R. Turner, Nelson Freimer, Ivona Pandrea, Cristian Apetrei

**Affiliations:** 1 Center for Neurobehavioral Genetics, Semel Institute for Neuroscience and Human Behavior, University of California Los Angeles, Los Angeles, CA USA; 2 Department of Molecular Genetics, Institute of Bioorganic Chemistry, Polish Academy of Sciences, Poznan, Poland; 3 Eye on Primates, Los Angeles, CA USA; 4 The Vatche and Tamar Manoukian Division of Digestive Diseases, Department of Medicine, David Geffen School of Medicine at UCLA, Los Angeles, CA USA; 5 Division of Gastroenterology, Hepatology and Parenteral Nutrition, VA Greater Los Angeles Healthcare System, Los Angeles, CA USA; 6 UCLA Microbiome Center, David Geffen School of Medicine at UCLA, Los Angeles, CA USA; 7 Department of Anthropology, Boston University, Boston, MA USA; 8 Department of Sociology, Anthropology, and General Studies, American Public University System, Charles Town, WV USA; 9 Department of Orthopedic Surgery, University of Pittsburgh, Pittsburgh, PA USA; 10 Department of Genetics, University of the Free State, Bloemfontein, South Africa; 11 Department of Anthropology, University of Wisconsin–Milwaukee, Milwaukee, WI USA; 12 Department of Pathology, University of Pittsburgh, Pittsburgh, PA USA; 13 Division of Infectious Diseases, Department of Medicine, University of Pittsburgh, Pittsburgh, PA USA

**Keywords:** SIV, Microbiome, Proteobacteria, Succinivibrio, Acute infection, Primate

## Abstract

**Background:**

The microbiota plays an important role in HIV pathogenesis in humans. Microbiota can impact health through several pathways such as increasing inflammation in the gut, metabolites of bacterial origin, and microbial translocation from the gut to the periphery which contributes to systemic chronic inflammation and immune activation and the development of AIDS. Unlike HIV-infected humans, SIV-infected vervet monkeys do not experience gut dysfunction, microbial translocation, and chronic immune activation and do not progress to immunodeficiency. Here, we provide the first reported characterization of the microbial ecosystems of the gut and genital tract in a natural nonprogressing host of SIV, wild vervet monkeys from South Africa.

**Results:**

We characterized fecal, rectal, vaginal, and penile microbiomes in vervets from populations heavily infected with SIV from diverse locations across South Africa. Geographic site, age, and sex affected the vervet microbiome across different body sites. Fecal and vaginal microbiome showed marked stratification with three enterotypes in fecal samples and two vagitypes, which were predicted functionally distinct within each body site. External bioclimatic factors, biome type, and environmental temperature influenced microbiomes locally associated with vaginal and rectal mucosa. Several fecal microbial taxa were linked to plasma levels of immune molecules, for example, MIG was positively correlated with *Lactobacillus* and *Escherichia*/*Shigella* and *Helicobacter*, and IL-10 was negatively associated with Erysipelotrichaceae, Anaerostipes, Prevotella, and Anaerovibrio, and positively correlated with Bacteroidetes and Succinivibrio. During the chronic phase of infection, we observed a significant increase in gut microbial diversity, alterations in community composition (including a decrease in Proteobacteria/Succinivibrio in the gut) and functionality (including a decrease in genes involved in bacterial invasion of epithelial cells in the gut), and partial reversibility of acute infection-related shifts in microbial abundance observed in the fecal microbiome. As part of our study, we also developed an accurate predictor of SIV infection using fecal samples.

**Conclusions:**

The vervets infected with SIV and humans infected with HIV differ in microbial responses to infection. These responses to SIV infection may aid in preventing microbial translocation and subsequent disease progression in vervets, and may represent host microbiome adaptations to the virus.

Video Abstract

**Supplementary information:**

**Supplementary information** accompanies this paper at 10.1186/s40168-020-00928-4.

## Background

The microbiome is increasingly recognized as an important player in the transmission [1–6] and pathogenesis [7, 8] of infection with human immunodeficiency virus (HIV) in humans. HIV infection causes a rapid loss of CD4^+^ T cells in the gut, thus leading to epithelial barrier damage and microbial translocation from the gut to the circulation and contributing to chronic immune activation, inflammation, and eventually disease progression. In sexually transmitted HIV infections in humans, the composition of the genital microbiome at colonization sites influences the risk of transmission [1–5, 9]. Penile anaerobic dysbiosis characterised by growth of oxygen-intolerant taxa increases a risk of HIV infection in men [1]. In women, the healthy vaginal microbiome is typically dominated by *Lactobacillus* [10], and its imbalance characterized by an increased microbial diversity [3]or *Lactobacillus* deficiency [2] is a risk factor of HIV acquisition. Insights into the role of the microbiota in infection have been obtained mainly from progressive hosts, which typically develop immunodeficiency upon infection. These hosts are represented by humans, in which HIV has been present on a large scale for nearly two generations, and experimentally infected Asian macaques, which are a laboratory model of pathogenic SIV infection, but are not exposed to the SIV *in natura*. A different perspective can be obtained from several African nonhuman primate species, which show a nonpathogenic course of infection because they have been naturally infected with their species-specific SIVs over long evolutionary periods (e.g., vervet monkeys and sooty mangabeys), during which they are believed to have adapted to live with the virus [11, 12]. However, the impact of the microbiota on the pathogenesis of the nonprogressing SIV infections in natural hosts has not been characterized to date.

The vervet monkey (genus *Chlorocebus*) is an Old World monkey species native to sub-Saharan Africa, showing a range of genetic adaptations to climate and pathogens [13, 14], and the most abundant natural host of SIV. African vervets transmit SIV predominantly through heterosexual contact, which results in a high prevalence of the SIVagm infection especially among adults (36–57% in males and 78–90% in females) [11, 15, 16]. Altogether, these aspects make the vervet a major natural reservoir species for SIV. Vervets in Africa have evolved with SIV over at least several hundred thousand years and show a massive polygenic adaptation to viruses, including SIV [13, 15]. In wild vervet populations, chronically infected vervets typically show a benign course of infection and do not manifest the hallmarks of progression to immunodeficiency. During chronic infection, they maintain stable circulating levels of mucosal translation biomarkers, specifically sCD14 and lipopolysaccharide, thus indicating intestinal barrier preservation [15, 17–19]. They also maintain normal levels of systemic immune activation and inflammation biomarkers in chronic SIV infection [15, 16]. These observations prompt questions about the links between the role of the gut microbiome in mucosal integrity preservation and the prevention of chronic immune activation in SIV-infected vervets.

Both progressive infection in pathogenic hosts and benign infection in vervets result in a massive acute depletion of CD4^+^ T cells residing in the gut during the early acute stage of experimental infection [17]. However, vervets can partially restore CD4^+^ T cells in the gut during the chronic stage of infection [17, 20]. The transition from the acute to chronic phase of infection in vervets is also characterized by regained control over type I interferon-stimulated genes, which are transiently upregulated during early infection in the immune tissues [21]; resolution of immune activation; and immune recovery [22]. The ability to control some early responses to a nonprogressing infection appears to be one of the key differences between nonpathogenic and pathogenic infections. However, the relationship between the host microbiome modifications and the course of SIV infection has not yet been studied.

The SIV-related microbiome has been characterized in two African great ape species naturally infected with their species-specific SIV in the wild: SIVcpz-infected Tanzanian chimpanzees, which show a progressive pathogenic course of infection, and SIVgor-infected Western lowland gorillas, in which the effects of SIV infection on the health remain unknown. The pathogenic SIVcpz infection in chimpanzees, which leads to AIDS-like clinical signs and greatly increases the risk of death [23, 24], has been associated with destabilization of the gut microbiome [25, 26]. In SIVgor-infected gorillas, the gut microbiome remained stable [27]. The relationship between the body microbiota beyond the gut—for example, changes in vaginal microbiota, which have also been implicated in pathogenic transmission of HIV [3, 5, 9, 28]—and nonprogressive SIV infection in a natural host species have not been studied to date.

To shed light on the natural microbiome in a nonprogressing host, we characterized the effects of various biological factors, including natural SIV infection, on the gut and genital microbiota in South African vervet monkey populations massively infected with SIV. We observed that the gut microbiota in SIV-infected vervets showed increased alpha diversity, decreased abundance of the phylum Proteobacteria (particularly the genus *Succinivibrio*), decreased predicted abundance of genes in the bacterial invasion of epithelial cell pathway, and partial control of early SIV-induced alterations in the gut during the chronic phase of infection. Controlled mechanistic studies are needed to provide more robust support that microbial responses to SIV infection in wild vervet populations contribute to protection against disease progression and represent evolutionary adaptations to the SIV pathogen.

## Materials and methods

### Samples

Microbial samples from four body sites in *N* = 107 vervet monkeys (*Chlorocebus pygerythrus*) from diverse geographic locations representing the Indian Ocean Coastal Belt (*N* = 33) and Savanna (*N* = 12) biomes from the KwaZulu-Natal Province (KZN), Azonal Vegetation (*N* = 29), Savanna (*N* = 27), and Grassland (*N* = 5) biomes in the Free State Province (FS) and Albany Thicket (*N* = 1) biome in the Eastern Cape Province (EC) in South Africa were analyzed (Supplementary Figure 1) [29]. The samples were collected in FS in July 2010, in KZN in August and September 2010, and in EC in June 2011 that is after or in the end of mating season of South African vervets (April–June), which exact dates vary between regions [30, 31]. To characterize the gut microbiome, we used two sample types: fecal samples (*N* = 44) collected directly from the rectum, and rectal swabs (*N* = 103). To characterize the genital microbiome, we analyzed swab samples from the vagina (*N* = 51) and penis (*N* = 20) (Supplementary Table 1 and Supplementary Figure 2). The KZN province is situated on the East coast of South Africa along the Indian Ocean and has a warm coastal climate. The FS province is located inland, is at relatively high elevation, and has an arid climate. The coastal area of Eastern Cape is characterized by a mild climate. Many samples from the KZN and FS provinces were characterized, and differential abundance analysis was performed on the samples from these provinces. The samples were obtained from the UCLA Systems Biology Biosample Repository [32]. The sampled individuals were previously characterized with respect to SIV infection and related health parameters [15].

The sample collection procedures were as follows: samples were collected through a capture-release study during which the animals were individually trapped [33] and briefly sedated with Zoletil or Ketamine (10 mg/kg) so that blood, microbial samples, and other biomaterials could be collected. All monkeys received subdermal injection of a microchip with a unique ID identifier. Phenotypic data, including general health assessments, which were conducted through minimally invasive procedures, and age estimation based on dental eruption patterns, were also collected [32]. Then, the animals were released back into their natural habitats near their capture sites and rejoined their troops.

Before fecal collection from the rectum and rectal swab collection, the perineal area was cleaned of gross contaminants using a chlorhexidine solution. The fecal samples were collected from the rectum digitally with sterile gloves and sterile lubricant. Each sample was divided into three aliquots and placed in cryovials. Swab samples from the rectum, vagina, and penis were collected with regular sterile swabs (Copan, US). Rectal swabs were inserted into the rectal opening and gently rotated along the rectal walls to collect the microbes. Vaginal swabs were gently inserted into the vagina and rotated. Before swabbing the vagina, vaginal pH was measured by placing the pH strip (EMD Chemicals) into the vaginal orifice, pressing the strip against the vaginal wall for 5 s and then removing for a readout. Penile swabs were used to swab the length of the penis, especially the area under the corona glandis. Each swab sample was collected in duplicate and placed in an individual cryovial with 1.5–2 ml of RNAlater (Ambion). Then, the collected samples were placed in short-term storage at − 20 °C or long-term storage at − 80 °C.

For comparative analysis, we used published data from Caribbean vervets [34, 35].

### Host- and SIV-related variables

General biological variables, including sex, geographic location, and dental age according to criteria modified from reference [36], as well as the biochemical phenotypes relevant to SIV infection (biomarkers for systemic inflammation and immune activation as well as SIV diagnosis), were collected from the study animals [15]. The blood sampling for the SIV infection diagnosis was performed concomitantly with the sample collection for the microbiome assessments during the same trap-sample-release procedure. The circulating levels of inflammatory biomarkers were measured previously with a 29-plex monkey panel of immune signaling molecules, including 15 cytokines (IL-1β, IL-1RA, IL-2, IL-4, IL-5, IL-6, IL-10, IL-12, IL-15, IL-17, G-CSF, GM-CSF, IFNɣ, IP-10, and TNFɑ), 10 chemokines (CCL-11 [eotaxin], IL-8, CCL-2 [MCP-1], CCL-22 [MDC], MIF, CXCL-9 [MIG] and CCL-3 [MIP-1ɑ], CCL-4 [MIP-1β], I-TAC, CCL-5 [RANTES]), and 4 growth factors (EGF, FGF-basic, HGF, and VEGF) [15].

SIV infection was previously diagnosed from plasma samples in the sampled animals [15]. The animals were classified as SIV-infected or uninfected on the basis of their SIV-specific PCR results. The stage of infection was determined according to viral load (VL), which was measured with RT-qPCR in plasma. Because high levels of viral replication associated with negative serologies are characteristic of acute infection [37], acute infection was identified on the basis of high VLs (i.e., 10^6^ RNA copies/ml or more) combined with SIV seronegativity, whereas chronic infection was indicated by VLs below this threshold and detectable anti-SIV antibodies.

### Bioclimatic variables

We analyzed bioclimatic data available from the WorldClim data [38]: annual mean temperature (mean temperature), max temperature of warmest month, min temperature of coldest month (min temperature), annual precipitation, precipitation of wettest month, precipitation of driest month.

### Microbiome analysis

#### 16S rRNA Sequencing

DNA was extracted using the Powersoil kit as per the manufacturer’s instructions (MO BIO, Carlsbad, CA, USA). The V4 region of 16S ribosomal RNA was amplified and underwent paired end sequencing on an Illumina HiSeq 2500 (San Diego, CA, USA) as previously described [39]. The 254 base-pair reads were processed using the DADA2 package in R (version 3.5.2) as previously described [40]. The mean sequence depth per sample was 91,715 ± 30,241 (standard deviation). Amplicon sequence variants were removed if they were present in less than 15% of samples. Samples with a depth of less than 10,000 were excluded from the analysis (2 fecal samples and 1 vaginal sample). Alpha diversity (i.e., diversity within a sample) and beta diversity (differences in composition across samples) were calculated in QIIME [41] using genus-level data rarefied to the sample with the lowest sequence depth at 11,539 sequences.

#### Statistical analyses

For 16s rRNA sequencing data, alpha diversity metrics that included Chao1 (a metric for species richness) and Shannon Index (a metric that incorporates both species richness and species evenness) were computed using QIIME. The statistical significance of differences in alpha diversity metrics was calculated using ANOVA. Beta diversity, a metric of differences between samples, was calculated using the square root of the Jensen-Shannon divergence and visualized by principal coordinates analysis in R [42]. Univariate Adonis, a permutational analysis of variance, was performed using 10,000 permutations to test for differences in the square root of the Jensens-Shannon divergence across the following variables: specimen type, gender, province where animals were sampled, age category, and SIV status. Differential abundance at the genus level was evaluated using DESeq2 in R, which employs an empirical Bayesian approach to shrink dispersion and fit non-rarified count data to a negative binomial model [43]. Variables listed in the multivariate analysis of DESeq2 were the same variables listed above for the multivariate Adonis analysis. *P* values for differential abundance were converted to *q* values to correct for multiple hypothesis testing (< 0.05 for significance).

Enterotype and vaginotype clustering was performed by using the Partitioning around medoids (Pam) function of the “cluster” package in R similar to prior published works [44]. To access the optimal number of clusters, we used the Calinski-Harabasz index from the R package “clusterSim.” Validation of the cluster was performed utilizing the silhouette validation technique. Graphical interpretation of the clusters was visualized with principal component analysis.

We conducted association analysis by performing Spearman correlations between host immune biomarkers and the intestinal microbiome from the feces in 23 individuals (10 from FS and 12 from KZN). From this analysis, we excluded individuals at the acute stage of SIV infection, because our previous studies have shown that the levels of some inflammatory biomarkers in the blood plasma are elevated during acute but not chronic SIV infection. Therefore, for association studies, we limited our analysis to chronically infected individuals.

#### Random forests classifier

A random forests classifier to predict SIV status was created in R using the randomForest package (https://cran.r-project.org/web/packages/randomForest) with 1001 trees and mtry = 2 [45]. Features inputted into the random forest classifier were those amplicon sequence variants associated significantly with SIV status as determined by multivariate DESeq2 models. The accuracy of the random forest classifier was estimated using a 10-fold cross-validation.

#### Predicted metagenomics

Metagenomic data of each sample was inferred from 16S rRNA sequencing data by using PICRUSt 1.1.3 (http://picrust.github.io/picrust), a well validated tool designed to impute metagenomic data from 16S rRNA compositional data [46]. 16S rRNA sequencing data was inputted into PICRUSt and normalized by copy number using default parameters. Predicted bacterial genes were then categorized into functional pathways using the KEGG database. Differential abundance of predicted bacterial genes and pathways in predicted metagenes were identified using DESeq2 with *p* values adjusted for multiple hypothesis testing.

## Results

### Natural composition of the microbiome in African vervets

**Fig. 1:**
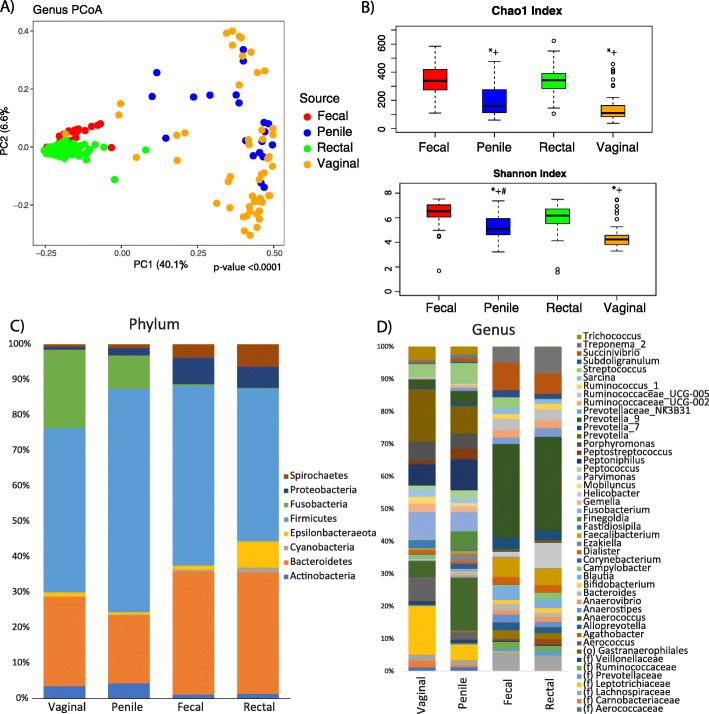
Characterization of the natural gut and genital microbiota in vervet monkeys. **a** PCoA, **b** alpha diversity, and taxonomic summaries at the **c** phylum and **d** genus levels across four body sites (fecal *N* = 44, rectal *N* = 103, penile *N* = 20 and vaginal *N* = 51). Firmicutes and Bacteroidetes are the most abundant bacteria in all sample types. Fusobacteria and Actinobacteria are common in the genital microbiome, and Spirochaetes and Proteobacteria are common in the gut microbiome. *Comparison versus fecal samples. +Comparison versus rectal samples. #Comparison versus vaginal samples. *+# *p* value ≤ 0.05

To understand the effects of SIV on the microbiome in a nonprogressing host of SIV in a natural environment, we studied the gut and genital microbial communities in the context of different geographic locations, developmental stages, sexes, and SIV infection in vervet monkeys in South Africa, by using 16S rRNA gene sequencing in 44 fecal, 103 rectal, 20 penile, and 51 vaginal microbiome samples (Supplementary Tables 1–2). The diversity of microbial communities differed by general body locations/sites (Fig. 1b) in African vervets. The gut microbiome from fecal and rectal samples showed higher alpha diversity than the genital microbiome from both the penis and vagina in vervets (*p* values < 0.001). Principal coordinate analysis (PCoA) indicated that the differences among samples were mainly driven by the general body site (Fig. 1a, Additional Data 1A). However, across all four body sites studied, Firmicutes and Bacteroidetes were the most predominant phyla in microbial communities (Fig. 1c).

### Fecal microbiomes showed a stratified structure in vervets

**Fig. 2:**
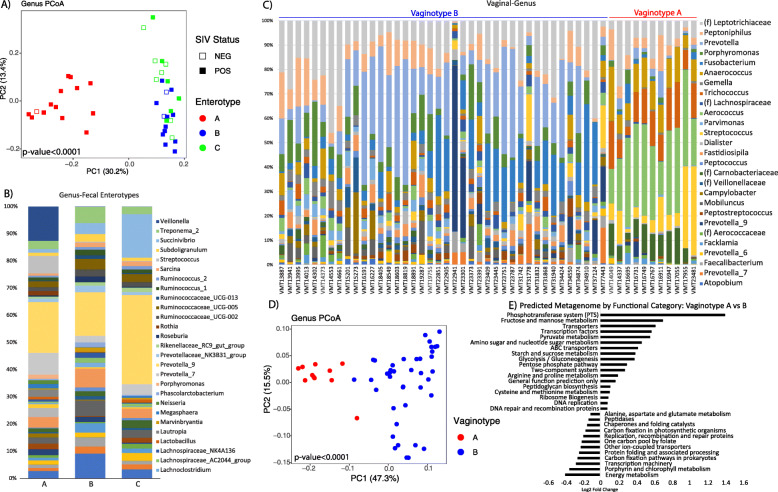
Bacteriological ecosystems in the gut and vaginal microbiomes in vervet monkeys. **a** Three enterotypes in fecal microbiome (*N* = 44) indicated by PCoA clustering and **b** their genus level taxonomic summaries. **c** Microbial profiles of the vaginal microbiome (*N* = 51) of individual vervet monkeys. **d** PCoA visualization of microbial compositional differences between the two vagitypes. **e** Differentially abundant microbial functional pathways between the two vagitypes (only top 30 abundant pathways represented)

In the gut microbiome (*N* = 44 fecal and *N* = 103 rectal samples), *Prevotella_9* was the most abundant genus in the fecal (20.9%) and rectal microbiomes (20.8%), and *Succinivirbrio* was the second most abundant genus in the feces (6.1%), whereas the abundance of other genera was below 6.1% (Fig. 1d). We observed only very low abundance of *Lactobacillus* in the gut (below 0.3% in the rectum and 0.3% in the feces), where this genus is known to play an important role in health in humans for its anti-inflammatory and gut protective functions. In the gut microbiome of African vervets, we distinguished three enterotypes with different functionalities (Fig. 2a, b). The A and B enterotypes were observed in adults only, whereas the C enterotype, which was associated with the expansion of *Prevotella*, was observed among younger individuals in addition to adults. The A enterotype differed from the B and C enterotypes in the predicted content of genes involved in energy metabolism and amino acid metabolism and synthesis (Supplementary Table 3). The strongest differences between the B and C phenotypes included “ABC transporter” and “porphyrin and chlorophyll metabolism” genes, which were overrepresented in the B enterotype.

### Vaginal microbiomes showed a stratified structure in vervets

In the vaginal microbiome (*N* = 51 vaginal samples), the most abundant taxa were the genus *Prevotella* (15%) (which, in humans, are involved in bacterial vaginosis and have proinflammatory properties promoting chronic inflammation and increasing the risk of acquiring HIV and other infections [47]) and the family of Leptotrichiacea (13.9%) (*Leptotrichia*, one of four genera within the family *Leptotrichiaceae*, ferments carbohydrates, thus producing lactic acid as its major metabolic end product [48]).

To examine the vaginal microbiome data from the African vervet populations in the context of those of other primates, we compared our data with those published by Yildirim et al. 2014 including Caribbean vervets from feral populations and a US-based research colony (Supplementary Figure 3, Additional Data 1B) [35]. In the vaginal microbiota in the Caribbean-origin vervets [35], two main community structures were apparent, when we inspected individual microbial profiles: one community structure was dominated by anaerobic *Sneathia* from the phylum Fusobacteria, and the other was dominated by facultative anaerobic *Aerococcus* from the phylum Firmicutes (Supplementary Figure 3C). In the South African vervets, we also observed a dichotomous pattern in the vaginal microbiota, yet of distinct composition than that in the Caribbean vervets. Through PCoA, we identified two clusters corresponding to two vagitypes in South African vervets: vagitype A, with a frequency of 21.6% dominated by four Firmicute genera *Anaerococcus*, *Trichococcus*, *Aerococcus*, and *Streptoccocus*; and vagitype B, with a frequency of 78.4% dominated by *Prevotella* and *Fusobacterium* (Fig. 2c, d). Vagitype A, compared with vagitype B, showed differential activity of numerous KEGG pathways (Fig. 2e). In vagitype A, the phosphotransferase system and the phosphotransferase system and fructose and mannose metabolism pathways showed the greatest increase, whereas energy metabolism and porphyrin and chlorophyll metabolism were the most decreased functions. We observed that the most enriched functional categories (phosphotransferase system, and fructose and mannose metabolism pathways) in vagitype A were associated with proliferative phase in humans, and the second most underrepresented functional category (the porphyrin and chlorophyll metabolism pathway) was associated with the secretory phase in humans [49]. These findings may suggest that the vagitypes represent a shift in the microbiome due to hormonal cyclicity. However, an ABC transporter pathway that was moderately enriched in vagitype A in vervets is strongly associated with the secretory phase pathway in humans, thus showing that there is no clear correspondence between the vervet vagitypes and human uterine phases.

Given that the vaginal pH can be associated with the composition of the vaginal microbiome and the estrogen levels [10], we measured vaginal pH (Additional Data 1B). The vaginal pH in vervets ranged from 5 to 8.5 with an average pH of 6.98 ± 1.27 (Supplementary Table 4). The near neutral vaginal pH in vervets is in contrast to the moderately acidic vaginal pH seen in most humans (average pH of 3.8–4.5) and is most likely the result of near absence of *Lactobacillus* in vervets. The vaginal pH did not have an effect on the overall bacterial diversity in the vervet vagina. However, acidic environment was associated with an increase in *Aerococcus*, *Trichococcus*, and *Streptoccocus* (characteristic to vagitype A), and a decrease in *Porphyromonas* (Supplementary Figure 4). In accordance with this observation, vagitype A was associated with more acidic pH (6.22 ± 0.712 mean pH ± Stdev), while vagitype B was associated with more alkaline pH (7.16 ± 0.68 mean pH ± Stdev) (*p* value 0.002).

### The effects of general biological variables on the composition and diversity of microbial communities in African vervets

We studied the effects on microbial community composition attributable to interindividual variation, sample source (body site), and fundamental biological variables often associated with disease epidemiology and health outcomes (namely, age, sex, and geographic origin) and assessed the proportion of variation attributed to these factors by using an Adonis analysis (Table 1). The analysis in a combined set of all samples from all body sites, including fecal (*N* = 44), rectal (*N* = 103), penile (*N* = 20), and vaginal samples (*N* = 51), showed that the major contributors to the variability in the composition of vervet microbial communities were interindividual differences (accounting for 34.74% of total variation, adjusted *p* value < 0.001) and sample type (accounting for 31.57% of total variation, adjusted *p* value < 0.001). We also observed significant effects (yet of smaller magnitude than those for ID and sample type) for other biological factors: developmental stage (3.69%, adjusted *p* value < 0.001), province (2.06%, adjusted *p* value < 0.001), and sex (1.07%, adjusted *p* value = 0.003, which is presumably mostly due to differences in genital microbiomes between males and females). We further explained the sources of the interindividual variation by using more specific information on animal age and place of origin. The individual effect dropped to 28.85% and the effect of geographic location (5.29%) and age (6.34%) increased when we used specific age categories (instead of broader developmental stages) and specific geographic sites (instead of provinces).

**Table 1:** Factors associated with microbial community composition based on Adonis analysis

Variable	*R*^2^ value	Unadjusted *p* value	Adjusted *p* value
**All samples**			
Developmental stage	0.037	0.002	< 0.001
Sex	0.011	0.018	0.003
Province	0.021	0.017	< 0.001
Source	0.316	< 0.001	< 0.001
Animal ID	0.347	< 0.001	< 0.001
Age category	0.053	0.008	< 0.001
SIV	0.011	0.043	0.18
**Rectal samples**			
Developmental stage	0.027	0.118	0.4012
Sex	0.010	0.147	0.326
Province	0.052	< 0.001	< 0.001
Age category	0.079	0.129	0.385
SIV	0.010	0.173	0.256
**Fecal samples**			
Developmental stage	0.076	0.463	0.4144
Sex	0.012	0.519	0.934
Province	0.051	0.442	0.418
Age Category	0.123	0.534	0.527
SIV	0.044	0.076	0.067
**Vaginal samples**			
Developmental stage	0.045	0.305	0.401
Province	0.155	< 0.001	< 0.001
Age category	0.055	0.091	0.201
SIV	0.015	0.339	0.413
**Penile samples**			
Developmental stage	0.100	0.051	0.028
Province	0.169	< 0.001	0.001
Age Category	0.136	0.141	0.218
SIV	0.035	0.479	0.656
**Acute/chronic samples**			
Source	0.418	< 0.001	< 0.001
Age category	0.015	0.118	0.297
Sex	0.004	0.288	0.356
Province	0.061	< 0.001	< 0.001

The effects of age (developmental stage or dental age category), sex, geography (province or geographic site), individual, body site, and SIV infection status on variation in microbial communities

### Geography-related variation in microbiota

Within each sample type (except for fecal samples), the communities tended to cluster by geographic location (Supplementary Figure 5A by province and Supplementary Figure 5B by geosite). Microbial community structures showed substantial variability due to province (Supplementary Figure 5C by province) and geographic site (Supplementary Figure 5D by geosite). Indeed, geography was the factor with the greatest effect on the overall microbiome composition within each sample type, except for feces. On the basis of an Adonis analysis, the effect of province was highest in genital samples (16.91% for penile and 15.51% for vaginal microbial variation) and lower in rectal samples (5.19%). Geographic locations were significantly associated with the microbial composition both in the vagina (32.64%) and rectum (18.39%). Importantly, these geographic factors remained significant when we controlled for other factors (age, sex, and SIV infection status). Given the effect of geography on microbiome composition, we attempted to identify microbial taxa driving this association (Supplementary Figure 5E, Additional Data 1C).

### Vervet microbiome and extrinsic environmental factors (geographic biomes and climatic variables)

We characterized the taxonomic composition of the vervet microbiome in relation to South African biomes inhabited by the vervets we studied (Supplementary Figure 6). The PCoA analysis of all samples showed that vervet microbiomes tend to group according to geographic biomes (*p* value < 0.0001). When each sample type was analyzed separately, the rectal and vaginal samples showed significant biome-related sample grouping (*p* value = 0.0002 and *p* value < 0.0001, respectively); however, there were no differences in microbial alpha diversity across different biomes (Supplementary Figure 7). Vagitype A was most associated with the grassland biome (70%), while B was associated mostly with the azonal vegetation and Indian Ocean coastal belt (77.5% combined) with *p* value < 0.001.

Two climatic variables, Mean Temp and Min Temp, were associated with significant differences in the microbiome composition while adjusting for sex, age, province, and SIV status (Additional Data 1D). Clustering of vaginal and rectal samples was associated with both Min and Mean Temps, and fecal sample clustering was associated with Min Temp (Supplementary Figure 8). Overall, Min Temp had the greatest changes, and vaginal samples were most affected by temperature. Several of the bacterial taxa associated with vagitype A (Streptococcus, Trichococcus, and Aerococcus) were enriched in the lowest tertile of annual Mean Temp *p* value < 0.001, and the mid tertile of Min Temp for the coldest month (*p* value < 0.0001), which comprised 90% of all samples with vagitype A (Supplementary Figure 9). Taken together, we observed that environmental temperature influences mostly the microbiomes associated with the local mucosa of outer body orifices with more exposure to environmental factors, while there is no such observable effect on the microbiome of fecal samples, which seem to represent a more “internal” microbiome.

### Sex-related and age-related variation in microbiota

Multivariate Adonis analysis did not indicate significant sex differences in the gut microbiota. However, differential abundance analysis revealed a significant increase in the genus CAG_352 of the phylum Firmicutes and decrease in the *Prevotella* genus of the Bacteroidetes in males in the rectum. In feces, the genus Paracaedibacteraceae of the phylum Proteobacteria was decreased in males (Supplementary Figure 10).

We characterized the microbial diversity and community profiles across the lifespan and compared the microbiomes of infants with that of adult individuals to identify potential microbial factors associated with early development (Supplementary Figure 11). We observed a decreased abundance of the genus *Dialister* of the phylum Firmicutes in infants in rectal samples when adjusting for sex, province, and SIV status. Low levels of *Dialister* were previously implicated in longevity in human populations [50].

### Links between the gut microbiome and systemic immune biomarkers

Host-microbiome interactions are important for both the local intestinal and extraintestinal homeostasis shaping the pathogenesis of inflammatory diseases [51]. To assess the natural homeostatic state between the gut microbiome and systemic inflammatory activity, we analyzed the association between the fecal microbiome composition and plasma levels of 29 inflammatory biomarkers in our vervet cohort. Nineteen immune biomarkers correlated with the abundance of at least one microbial genus (Supplementary Figure 12). The strongest association was observed for MIG, which was positively correlated with *Lactobacillus*, followed by *Escherichia/Shigella* and *Helicobacter*. IL-10 was associated with several bacteria; for example, it was negatively correlated with Erysipelotrichaceae, *Anaerostipes*, a member of Ruminococcaceae, *Prevotella* and *Anaerovibrio*, and was positively correlated with Bacteroidetes, *Succinivibrio*, and a member of Ruminococcaceae. Our observations demonstrated that the gut microbiome is closely linked to the systemic inflammatory biomarkers in apparently healthy individuals.

### Alterations in body microbiota associated with SIV infection

To shed light on the potential links between the microbiota and a typically nonprogressive course of SIV infection in vervets, we analyzed the association of gut and genital microbial communities with SIV status by using a cross-sectional sample set. We compared the microbiomes between SIV positive and SIV negative vervets, and then between uninfected vervets and a subset of infected vervets determined to be acutely infected or chronically infected, on the basis of previously performed diagnoses in the blood samples collected simultaneously with the samples for the microbiome studies [15] (Supplementary Figure 13).

**Fig. 3:**
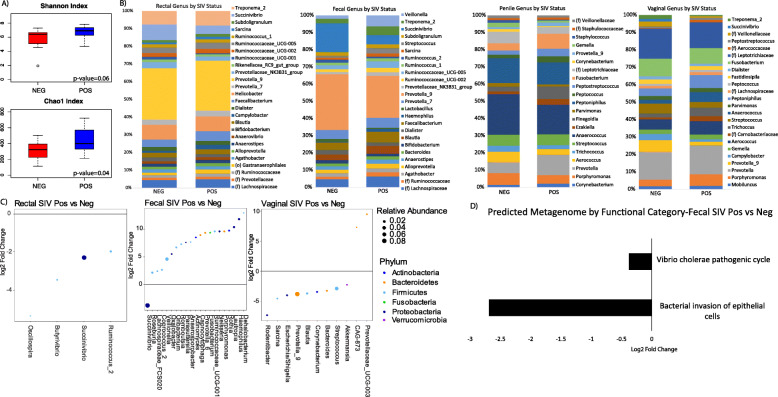
SIV infection is associated with higher microbial diversity and altered microbiome composition and function. Characterization of SIVpos and SIVneg samples (respectively, 62 and 41 from the rectum, 33 and 11 from the feces, 11 and 9 from the penis, and 41 and 10 from the vagina) with respect to **a** alpha diversity in feces, stratified by SIV status and **b** community structure for four body sites stratified by SIV positive/negative status. **c** Differentially abundant genera between SIVpos and SIVneg individuals at all body sites (There were no differentially abundant genera in penile samples). Analysis was adjusted for age, sex, and vervet location. **d** Functional pathways associated with SIV infection in the predicted metagenome of fecal samples

We assessed microbial alpha diversity across four sample types in relation to SIV infection status (SIV positive vs. SIV negative). SIV-infected vervets showed a significant increase in fecal microbiota richness (*p* = 0.02, Chao1 index) and a trend toward increased diversity by the Shannon index (*p* = 0.06), whereas such effects were not observed in rectal, penile, or vaginal microbiota (Fig. 3a).

We compared the microbiome compositions between the SIV-infected (*N* = 146) and uninfected individuals (*N* = 71) in rectal samples (62 SIV positive and 41 SIV negative), fecal samples (33 SIV positive and 11 SIV negative), penile (11 SIV positive and 9 SIV negative), and vaginal samples (41 SIV positive and 10 SIV negative) (Fig. 3b). The group differences between SIV positive and SIV negative vervets were statistically significant in a combined set of samples from all body sites (unadjusted *p* value = 0.043, *R*^2^ = 0.011, Adonis) and approached statistical significance in the fecal samples (*p* value = 0.067, *R*^2^ = 0.044, Adonis, adjusted for developmental stage, sex, province, and age category). Fecal (but not rectal) samples tended to cluster by SIV infection status (Fig. 2a). There was a difference in the fecal enterotype distribution between the SIV-infected and uninfected individuals (chi-square test *p* value = 0.012). Enterotype A consisted of 92.9% of SIV-infected individuals, enterotype B consisted of 84.6% of SIV-infected individual, while enterotype C only consisted of 46.2% of SIV-infected individuals. Among the SIV-uninfected individuals, enterotypes A and B appeared rarely, while enterotype C was more common. We also compared the relative abundance of individual taxa in the gut microbiome between SIV-infected and uninfected monkeys. During SIV infection, we identified two genera significantly differentially abundant in the gut microbiome after adjustment for sex, age, and geographic location: *Succinivibrio* and *Veillonella* (Fig. 3c). In SIV-infected vervets, the *Succinivibrio* genus of Proteobacteria was underrepresented in both the fecal and rectal microbiomes. *Succinivibrio* was the dominant genus in the phylum Proteobacteria in the vervet gut microbiome. (*Succinivibrio* was present across all sample types but appeared overabundant in the rectum and fecal microbiome compared with other body sites). During SIV infection, the *Veillonella* genus of Firmicutes was overabundant in the fecal microbiome. The Veillonellaceae family was present across all sample types (yet at low abundance) and was enriched in the genital compared with gut microbiome in vervets.

To identify microbial metabolic pathways in the gut associated with SIV infection, we assessed functional profiles in the predicted metagenomes in fecal samples from SIV positive and SIV negative individuals. “Bacterial invasion of epithelial cells” and “Vibrio cholerae pathogenic cycle” KEGG pathways were significantly lower in SIV-infected than uninfected individuals (Fig. 3d). These observations suggest that the altered composition of the gut microbiome during SIV infection can influence the risk of acquisition and reproduction of environmental pathogens.

In vervets, we did not observe significant SIV-related variation in the abundance of *Lactobacillus*, which appears to be a key regulator of immune homeostasis in pathogenic SIVmac infection in macaques [52] and has been implicated in HIV pathogenesis as a potential protective factor [7, 8]. In untreated HIV-infected patients, *Lactobacillus* has been positively associated with higher CD4^+^ T cell count, lower viral loads, and decreased microbial translocation [7]. The relative abundance of *Lactobacillus* in vervet feces (0.005 and 0.012 in SIV-infected and uninfected vervets, respectively) was generally lower than that observed in humans.

In the genital microbiome, SIV infection was associated with significant abundance shifts of microbial taxa only in the vagina. Among the more abundant bacteria, the *Prevotella_9* genus of the phylum Bacteroidetes and the *Streptococcus* genus of the phylum Firmicutes were underrepresented in SIV-infected individuals. We did not observe significant differences in the vaginal pH between SIV-infected and SIV uninfected females.

### Microbial differences between acute and chronic phase SIV infection

To investigate whether early microbiome responses to infection differ from those in the chronic state, we examined microbial profiles in individuals who were uninfected (*N* = 71), acutely infected (*N* = 23) or chronically infected (*N* = 104) based on the fecal samples (11 SIV negative, 4 acutely infected, 23 chronically infected), rectal samples (41 SIV negative, 11 acutely infected, 43 chronically infected), vaginal samples (10 SIV negative, 6 acutely infected, 30 chronically infected), and penile samples (9 SIV negative, 2 acutely infected, 8 chronically infected). The number of individuals acutely infected with SIV is relatively small because the acute SIV infection, characterized by the peak of SIV replication in the blood plasma prior to seroconversion (according to the Fiebig II stage [37], can be detected for a relatively short period of time, approximately 2–4 weeks following infection.

**Fig. 4:**
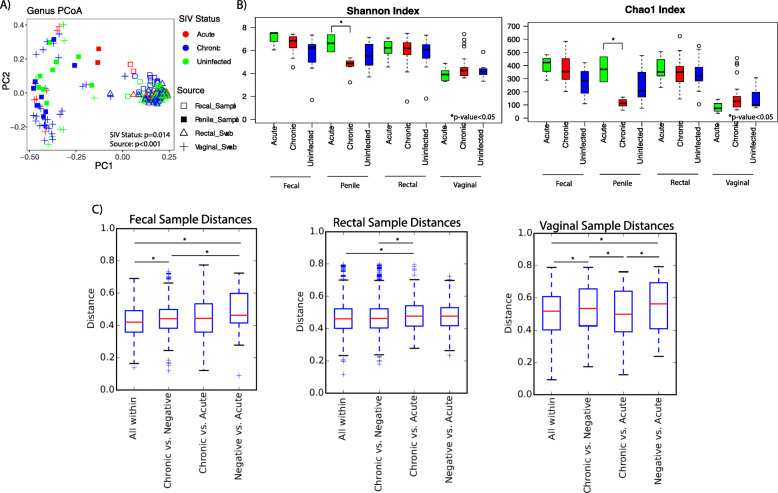
Microbiome across stages of SIV infection in vervets. **a** PCoA colored by stages of SIV infection. *P* values are adjusted for collection site, gender, and age. **b** Alpha diversity as presented by Shannon index across different body sites and SIV stages. **c** Distance box plots comparing distances within a particular SIV stage (i.e., all within) and between SIV infection states across different sample types. The first boxplot contains the distances between all samples that are within the same category, i.e., within negative samples, within acutely infected samples and within chronically infected (all within). The subsequent boxplots represent the distances between all samples in different categories: all chronic samples vs all negative samples, all chronic samples vs. all acute samples, and all negative samples vs. all acute samples. The microbiome samples comprise of the fecal samples (11 SIV negative, 4 acutely infected, 23 chronically infected), rectal samples (41 SIV negative, 11 acutely infected, 43 chronically infected), vaginal samples (10 SIV negative, 6 acutely infected, 30 chronically infected), and penile samples (9 SIV negative, 2 acutely infected, 8 chronically infected). *Indicates comparisons with a *p* value < 0.05

The penile, rectal, and fecal microbiomes showed a trend toward increased microbial diversity in acute phase individuals compared with uninfected and chronically infected individuals, and for the penile microbiome, this difference reached statistical significance (Chao1 index *p* value = 0.001, Shannon index *p* value = 0.008) (Fig. 4). Among the four studied sample types, the penile microbiota also showed a separation by acute and chronic infection stage in beta diversity analysis (Fig. 4). However, extreme caution in interpretation of these data is warranted because of the small number of penile samples from acutely infected individuals (*N* = 2). The animals from which we collected the samples belong to different troops but are from the same general location (the Sandveld Nature Reserve in the Free State Province). We cannot completely exclude the possibility that other factors, environmental or genetic, can bias this observation.

We explored shifts in the relative abundance of individual microbial taxa between chronic and acute phase of SIV infection using DESeq2 analysis adjusted for sex, age, and geographic location (Supplementary Figure 14). When chronic infection was compared with the acute infection, the following differences were observed: an overrepresentation of *Ruminococcaceae_UCG-0009*, a member of the genus *Ruminococcaceae* (known for its anti-inflammatory properties and role of gut homeostasis) from the phylum Firmicutes in the fecal microbiome; the genus *Butyricicoccus* of the phylum Firmicutes in the rectal microbiome; and *Muribaculaceae* and *Rikenellaceae RC9 gut group* from the phylum Bacteroidetes, *VadinBE97* from the phylum Lentisphaerae, and *Clostridiales vadin BB60 group* and Ruminococcaceae NK4A214 group from the phylum Firmicutes in the vaginal microbiome.

To uncover global relationships among different infection states (SIV negative, chronic infection, and acute infection), we analyzed the distances within particular states (i.e., All within) and the distances between different states for fecal, rectal, and vaginal samples, where the value of each grouping is measured as the distances between samples. The results are represented in the distance boxplots (Fig. 4c). For fecal samples, we observed a significantly higher difference between the “chronic vs. negative” distance vs. the “negative vs. acute” distance (*p* value< 0.05), indicating that the differences between chronic and negative samples are less important than the differences between acute and negative samples. We also inspected heat-maps of microbial community composition across different infection states. We observed that, consistently across all four body sites, the microbiome in uninfected individuals clustered together with that in chronically infected than acutely infected individuals (Supplementary Figure 15). Taken together, these observations suggest that some changes in the fecal microbiome occurring during the acute phase of the SIV infection normalize after the transition to chronic phase. However, longitudinal studies under controlled conditions are necessary to prove the hypothesis that SIV-induced alterations in the microbial communities during the acute phase are partly transient and come under control during the chronic phase.

One of the microbes that did not follow this pattern was Succinivibrio, which showed a progressive decrease to the lowest levels in chronic phase in the fecal and rectal microbiomes (which showed the lowest levels in chronic phase) (Supplementary Figure 15).

### The gut microbiome as a predictor of SIV infection

**Fig. 5:**
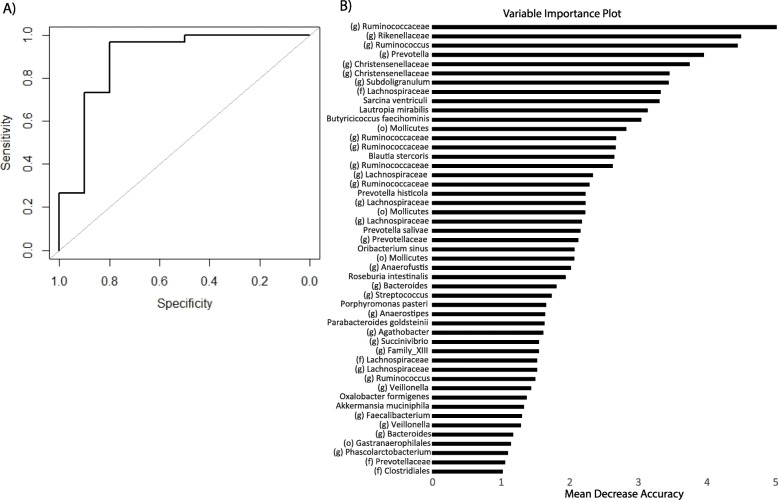
SIV infection classifier based on the fecal microbiota (*N* =44). **a** ROC curve with AUROC of 0.95, sensitivity 0.5, and specificity 0.97 and **b** most important taxa for the predictor. Variables with higher mean decrease accuracy have a greater contribution to the accuracy of the classifier

Fecal microbiome can serve as a proxy for the microbiome of the gut, i.e., the site of SIV/HIV pathogenesis. We employed fecal samples to detect microbial profiles characteristic to SIV infection. The association of gut microbiota composition with SIV infection created an opportunity to develop a classifier of SIV infection based on fecal microbiome composition. We developed a random forests classifier for SIV infection status using species level abundances in the fecal microbiome that had high accuracy (Fig. 5). The area under the receiver operating curve (AUROC) was 0.95 with high specificity (0.97) and moderate sensitivity (0.50). Among the ~ 50 markers selected by the classifier, the most important bacterial species were members of the families Ruminococcaceae and Rikenellaceae, and the genus *Ruminococcus*.

## Discussion

### The natural body microbiome of the vervets

In African vervet populations, the compositions of microbial communities are shaped primarily by sample type (body location) and interindividual variation, followed by geography, with minor effects of age and sex. External bioclimatic factors (biome type and temperature) influence microbiomes locally associated with vaginal and rectal mucosa in the vervet. However, we did not observe such an effect in fecal samples, which predominantly contain luminal bacteria carried through different parts of the gastrointestinal tract and that are expected to be closely linked to the host diet [53]. This observation suggests that environmental factors mainly shape microbiome of outer orifices, which have greater exposure to the bioclimatic factors, and probably through other factors than host diet.

The gut microbiomes from fecal and rectal samples showed higher alpha diversity than did the genital microbiomes from penile or vaginal samples. In humans, similarly, alpha diversity is lower in the vagina than the gut, but this pattern is mostly driven by the extreme dominance of *Lactobacillus* in the vaginal ecosystem and an overall very low diversity of the microbiome in the vagina (which appears to be a human-specific trait) [54], whereas in vervets, all body sites studies showed marked diversity. The low abundance/lack of *Lactobacillus* in the vaginal microbiome of vervets is accompanied by increased vaginal pH, in contrast to typically acidic pH of the *Lactobacillus*-rich environment of the human vagina [10].

The fecal and vaginal microbiome of the vervet show stratification into functionally distinct ecosystems: three gut enterotypes in fecal samples and two vagitypes; an effect that was not observed in rectal or penile microbiomes. Several factors were associated with different vagitypes including vaginal pH, which can be potentially indicative of estrogen production regulated through cycle- or age-related ovarian activity [55], and interconnected ecological factors—environmental temperature and biome type. To what extent these factors are associative or causative of the stratification of vaginal microbiome, and whether or not other factors contribute to this phenomenon could be answered through longitudinal studies in the future.

The vaginal microbial community in South African vervets (*Ch*. *pygerythrus*) differed from that in the Caribbean-origin vervets (*Ch*. *sabaeus* vervet native to West Africa) (Additional Data 1B). Thus, South African vervets display two vagitypes, neither of which shows clear dominance by individual genera, whereas Caribbean-origin vervets generally show two distinctive patterns in the vaginal microbiome, one dominated by *Sneathia* and the other dominated by *Aerococcus*. We did not observe an analogous effect in the rectal microbiome in our South African vervets and Caribbean vervets characterized in Amato et al. 2015 (Supplementary Figure 16).

Under healthy conditions, the composition of the microbiota residing in the vervet gut is associated with systemic immune markers. The molecules most strongly linked to microbial abundance (MIG and IL-10) are known for their roles in gut immune homeostasis. MIG is involved in the antimicrobial response in the intestinal mucosa [56, 57], and IL-10 is known to play a major role in suppressing proinflammatory activities in the gut [58].

### SIV-associated increases in fecal microbiota diversity

In African vervets, we observed an increased microbial diversity in the fecal microbiota with SIV infection, in contrast to HIV-associated loss of microbial diversity reported in several human studies, e.g., [59–62]. However, some studies have reported no such associations, e.g., [8, 63, 64] or even a decrease in microbial diversity in HIV-infected subjects [65]. The lack of a consensus among microbiome studies in HIV-infection may be the result of difficulties in controlling for numerous confounding factors influencing HIV-related microbiome studies (i.e., geographic environment, antibiotics, antiretrovirals and other treatments, sexual practices, and sampling methods) [66]. However, several human studies have linked the loss of microbial diversity to biomarkers of disease progression. Microbial diversity in the gut in HIV-infected subjects is highly negatively correlated with microbial translocation and monocyte activation markers [67], and positively with CD4^+^ T cell count [60, 68, 69]. Decreased microbial richness is a strong predictor of complications in HIV-infected subjects [70]. Given that these observations imply a loss of microbial diversity as a driver of HIV pathogenesis, the corollary of the increased microbial diversity in the gut observed in SIV-infected vervets is that microbial diversity in the gut may play a role in preventing microbial translocation and chronic immune activation and thus may have beneficial anti-immunodeficiency effects.

To assess the gut microbiota of vervets in relation to pathogenic hosts, we compared our results to phylum-level data from rectal swabs from humans and macaques [54] In all three species, Bacteroidetes, Firmicutes, and Proteobacteria are the most dominant phyla (relative abundance > 2%), while African vervets specifically had higher abundances of the phyla Epsilonbacteraeaota and Spirochaetes.

### Alterations in gut microbial composition with SIV infection

HIV infection in humans is characterized by several consistently observed changes in the abundance of microbial taxa in the gut, some of which are correlated with predictors of HIV disease progression. For example, HIV infection in humans correlates with an expansion of species belonging to the Proteobacteria phylum [8, 59, 63, 71, 72], which is linked to lower CD4^+^ T cell counts [60]. In vervet monkey populations, the most striking alteration associated with SIV-infection, observed in both the fecal and rectal microbiomes, is underrepresentation of the phylum Proteobacteria, particularly the genus *Succinivibrio*. In contrast to the gut microbiome in SIV-infected vervets, in the intestinal microbiota in fecal samples from HIV-infected individuals, *Succinivibrio* is overabundant and can be used as a biomarker of HIV infection [68]. Members of the Succinivibrionaceae family are involved in the transport and accumulation of anti-inflammatory molecules and viral inhibitors, and may play a role in the accumulation of proinflammatory molecules in the gut [73].

Expansion of the phylum Proteobacteria has been implicated in various developmental and health states such as decreased stability of the gut microbiome (characteristic of the normal neonatal stage), and metabolic diseases and gut inflammation, and it is considered a diagnostic biomarker of dysbiosis and disease risk [74]. In HIV-infected individuals, the expansion of the Proteobacteria phylum in the gut is also associated with dysbiosis [59, 63], and in chronically SIV-infected macaques, Proteobacteria preferentially translocate from the lumen and accumulate in peripheral tissues, where their relative amounts are positively correlated with the proportion of activated CD4^+^ T cells [75]. These findings suggest that these bacteria may play a role in pathogenesis. In contrast, the family of Succinivibrionaceae is underabundant in the porcine colon during nematode infection, where it has been suggested to play a protective role against gut inflammation [76]. The decrease in the abundance of *Succinivibrio* in the intestinal microbiota of SIV-infected vervets is a change in the opposite direction from that observed in HIV-infected subjects but the same as that observed in porcine nematode infection (which can suppress proinflammatory responses). Together, these findings may suggest that the decrease in the relative abundance of *Succinivibrio* in SIV infection may help to maintain the mucosal barrier and prevent chronic immune activation in a natural host; however, this potential protective mechanism needs to be validated through functional studies.

Another significant alteration in the gut microbiota of SIV-infected African vervets was overrepresentation of the genus *Veillonella* of the phylum Firmicutes in the feces. *Veillonella* is known to be part of the normal flora of the oral, genitourinary, respiratory, and intestinal tracts. In the human gut microbiome, the abundance of *Veillonella* is significantly elevated in stool samples from people with IBD and Crohn’s disease, and correlated with metabolites associated with Crohn’s disease [77]. Lentiviral infection in vulnerable hosts is associated with changes in the abundance of *Veillonella* in the oral and vaginal ecosystems. *Veillonella* are enriched in the oral microbiome in SHIV-infected cynomolgus macaques that developed COPD [78] and in HIV-infected subjects compared with HIV-uninfected controls in the oral microbiome [79, 80]; in addition, *Veillonella* is associated with increased vaginal inflammation and a risk of HIV acquisition [2]. The increase in *Veillonella* in SIV-infected vervets may represent a shared component of the lentiviral infection in susceptible and adapted hosts or may suggest that *Veillonella* is not playing an important role in the pathogenesis of these diseases and it is probably a consequence rather than a cause of intestinal lesions.

The functional pathways in the fecal microbiome altered during SIV infection in vervets are directly involved in microbial pathogenesis. The functional profiles of SIV-associated microbiota in vervets showed a significant underrepresentation of the “Bacterial invasion of epithelial cells” pathway. The epithelial cells in the gut mucosa are the route of entry of bacterial pathogens and respond to bacterial invasion by upregulation of pro-inflammatory factors and cell apoptosis [81]. Under normal conditions, pathogenic bacteria invading the gut epithelium are eliminated through phagocytosis in the lamina propria and mesenteric lymph nodes, but in HIV/SIV-infected immunocompromised vulnerable hosts, bacteria translocate beyond the intestine to other internal organs [82]. A persistent pathogenic translocation of gastrointestinal microbial products into the circulation has been proposed as a major driver of the chronic immune activation associated with immunodeficiency and HIV/SIV disease progression [75, 83]. To what extent the decreased bacterial cell invasion in the gut microbiome during SIV-infection in vervets may contribute to the lack of intestinal inflammation and cell loss, and thereby to reduced microbial translocation outside of the intestines in this well-adapted host, requires further investigations. Consistently, we did not observe differences in microbial translocation biomarkers (sCD14 and lipopolysaccharide) between SIV-infected and uninfected vervets in our previous studies, thus suggesting maintenance of the gut mucosal barrier [15, 16]. Downregulation of another pathway, “Vibrio cholerae pathogenic cycle,” in the gut in SIV-infected vervets stays in contrast to the overrepresentation of this pathway in HIV-infected subjects on ART [69]; however, mechanistic studies are needed to explore whether the reduction of this pathway may have a beneficial effect.

Another microbiome feature of the natural SIV infection in South African vervets is that the fecal microbiome of the uninfected monkeys was more different from that of the acutely infected individuals than from chronically infected individuals, suggesting partial transient/temporary shifts in microbial communities in the fecal microbiome during acute infection. While this observation requires further validation, it appears inline with a distinctive feature of nonprogressive SIV infection in vervets compared with the progressive infections in susceptible hosts, which is the ability to recover in chronic phase from several pathogen-driven responses during acute infection. Chronically infected vervets partially replenish CD4^+^ T cells depleted during acute infection in the gut [17] and normalize the expression of interferon-stimulated genes upregulated in acute phase [21], and our results suggesting similar partial normalization of the fecal microbiome composition during chronic phase of SIV infection may in part by related to these adaptive processes.

### Genital microbiome and SIV transmission

Beyond the gut, SIV-infection was associated with microbial variations in the genital microbiome. In humans, the genital microbiome has been implicated in shaping the risk of HIV transmission [1, 2, 84], raising the question of whether such a link exists in NHPs such as the vervet. South African female vervets showed markedly higher prevalence of SIV infection than males [15], suggesting the existence of factors modulating sex-specific differences in susceptibility to acquisition of the virus. This phenomenon could be attributable to multiple factors, including lower transmissibility of SIV through foreskin than vaginal mucosa [85], differences in SIV exposure between males and females (which can result from differences in male reproductive access based on social rank [86] or factors influencing female mate choice [87]), or genital microbiome. Given that genital dysbiosis contributes to HIV transmission [2, 84], the observation of increased microbial diversity in the penile microbiome of acutely SIV-infected vervet males can hypothetically be a contributing factor to greater transmissibility of the virus to females. However, this observation was obtained from a small number of penile microbiome samples from acutely infected males (*N* = 2), and, as such, can only motivate a suggestion for a potential role of penile microbiome in SIV transmission. Confirmation of this scenario would require longitudinal studies or experimental SIV challenge to test the hypothesis of the potential role of penile microbiome in SIV transmission.

### SIV diagnosis based on fecal microbiome composition

Many field studies in wild NHP populations often perform extensive noninvasive sampling involving collection of stool samples from the ground. Such specimens are feasible to collect longitudinally and at a large scale, thus minimizing the effects on wild NHPs. We propose that the fecal microbiome can serve as a source of noninvasive biomarkers of SIV infection and therefore can provide important information about the health of monkey populations beyond the microbiome.

## Conclusions

Together, our findings highlight several distinctive features of nonpathogenic SIV infection in wild vervets, including increased microbial diversity, compositional shifts (especially a decrease in *Succinivibrio* and Proteobacteria), decreases in pathways associated with infection with pathogenic microbes, and greater similarity of microbial patterns between the uninfected state and the chronic phase rather than the acute phase of infection in the gut. Although it is tempting to speculate that the SIV-associated changes of the gut microbiome in the vervet are driving a nonprogressive course of SIV infection in this species, a mechanistic validation and wider studies, including longitudinal sampling and controlled microbiome manipulations, in the wild populations and in the research colonies of vervets are needed to evidence the modulatory role of the gut microbiome in SIV infection. The vervet is the first natural host species of SIV, for which microbiome was characterized in a context of natural SIV infection. We anticipate that characterizing microbiomes of other natural hosts would help to validate or refute these hypotheses.

## Supplementary information

**Additional file 1 : Supplementary Figure 1**. Geographic origins of study animals. **Supplementary Figure 2**. Maps of sampling sites. The maps were generated using GPS Visualizer [90] with Google Hybrid Map as a background using Map data © 2020 AfriGIS (Pty) Ltd Imagery© 2020 NASA, TerraMetrix. **Supplementary Figure 3**. Vaginal microbiome in South African vervets in our study compared to Caribbean-origin vervets and other NHPs based on the Yildrim et al. 2014 data [35]. (A) Average community structure in South African vervets (by SIV status), Caribbean origin vervets and other NHPs [35]. Species demarcation: African vervets (SIV Pos and SIV Neg), free-ranging Caribbean vervets (Vw), captive Caribbean-origin vervets (Vc), mangabeys (M), red colobus (Rc), yellow baboon (Bab), olive baboon (Bc), chimpanzee (Chimp), humans (H), lemurs (L), black howler (Bh). (B) Microbial community structure per individual in South African vervets, (C) Community structure per individual in Caribbean-origin vervets [35]. **Supplementary Figure 4**. A) PCoA of beta-diversity colored by vaginal pH. Acidic pH is a pH <7 while an alkaline pH is a pH > 7. B) Chao1 and Shannon Index of vaginal samples pH. C) Genus taxonomic plots of vaginal samples by pH. D) Differentially abundant microbial genera in acidic vaginal samples vs. alkaline vaginal samples. **Supplementary Figure 5**. Geography-related microbiome. (A) PCoA for each body site clustering by province. (B) PCoA for each body site clustering by geographic site. (C) Community structure by province. (D) Community structure by geographic site. Differentially abundant taxa between KZN and FS in (E) rectal and (F) fecal microbiome. **Supplementary 6**. Genus taxonomic plots of D) fecal, E) rectal, F) penile, and G) vaginal samples by the different biomes. **Supplementary Figure 7**. PCoA of beta-diversity, Chao1 index and Shannon index by different biomes for (A-C) all samples, (D-F) fecal samples, (G-I) rectal samples, (J-L) penile samples, and (M-O) vaginal samples. **Supplementary Figure 8**. PCoA plots of beta-diversity and alpha diversity metrics of climate variables. PCoA plots of annual mean temperatures in tertiles (Mean Temp Tertile) and minimum temperature of the coldest month in tertiles (Min Temp Tertile) for all samples (A,B), fecal samples (C,D), rectal samples (E,F), penile samples (G,H), and vaginal samples (I,J). Chao1 and shannon index did not differ by any sample type except for Mean Temp Tertile in penile samples (K,L). *Represents p-value<0.05 between either lowest or mid tertile vs the highest tertile. **Supplementary Figure 9**. Genus taxonomic plots of the annual mean temperatures in tertiles (Mean Temp Tertile) and minimum temperature of the coldest month in tertiles (Min Temp Tertile) by sample type: (A,B) Feces, (C,D) Rectal, (E,F) Penile, (G,H) Vaginal. Only genera with greater than or equal to 1% abundances is shown *Represents the genera that were statistically different between the highest tertile and the lowest tertile adjusting for multiple comparison. **Supplementary Figure 10**. Sex-related gut microbiome. Differentially abundant taxa in males compared to females in feces (A) and in rectum (B). **Supplementary Figure 11**. Age-related microbiome. Microbial community structure for each body site. A) Rectal, C) Penile, D) Fecal, E) Vaginal. B) Differentially abundant genera between the rectal microbiome in infants and adults. **Supplementary Figure 12**. Association of immune biomarkers with microbial taxa in fecal microbiome. **Supplementary Figure 13**. PCoA of microbial taxa in all samples colored by SIV positive/SIV negative status. **Supplementary Figure 14**. Differentially abundant microbial genera in chronic vs. acute SIV infection by body site. Analysis was adjusted for sex, age, and vervet location. Penile samples did not show any genera that were significantly different between acute and chronic SIV states after adjusting for other covariates. **Supplementary Figure 15**. Microbial composition (depicted by heat maps) in SIV negative, SIV acutely infected and SIV chronically infected individuals at four body sites. Microbiome samples comprise of fecal samples (11 SIV negative, 4 acutely infected, 23 chronically infected), rectal samples (41 SIV negative, 11 acutely infected, 43 chronically infected), vaginal samples (10 SIV negative, 6 acutely infected, 30 chronically infected), and penile samples (9 SIV negative, 2 acutely infected, 8 chronically infected). Only genera with a relative abundance > 0.5% are shown. **Supplementary Figure 16**. Comparison of rectal microbiome community structure between vervets from Caribbean populations and Caribbean-origin captive vervets on Western-style diet (based on data from Amato et al. 2015 [34]) and SIV negative and SIV positive vervets from South Africa.

**Additional file 2 : Supplementary Table 1**. Characteristics of microbial samples and individuals used in the studies. **Supplementary Table 2**. Relative abundances at the phylum and at the genus level within the bacterial communities in four sample type. Showing taxa with > 1% relative abundance in at least one sample type. **Supplementary Table 3**. Relative abundances of predicted metagenome categorized by function across fecal enterotypes. Only showing genes with significant differential abundance between the enterotypes and at a relative abundance > 0.1%. Data is expressed as relative abundance (%). **Supplementary Table 4**. Vaginal pH.

**Additional file 3 Additional Data 1.**

## Data Availability

The microbiome dataset comprising of raw 16S rRNA sequences generated during the current study was deposited under the National Center for Biotechnology Information BioProject PRJNA603995 (https://www.ncbi.nlm.nih.gov/bioproject/603995).
