# Supplementary material for: Shifts in microbial diversity, composition, and functionality in the gut and genital microbiome during a natural SIV infection in vervet monkeys
Source: Microbiome. 2020 Nov 6;8:154. doi: 10.1186/s40168-020-00928-4 (PMC7648414; doi:10.1186/s40168-020-00928-4)

Supplementary Figure 1

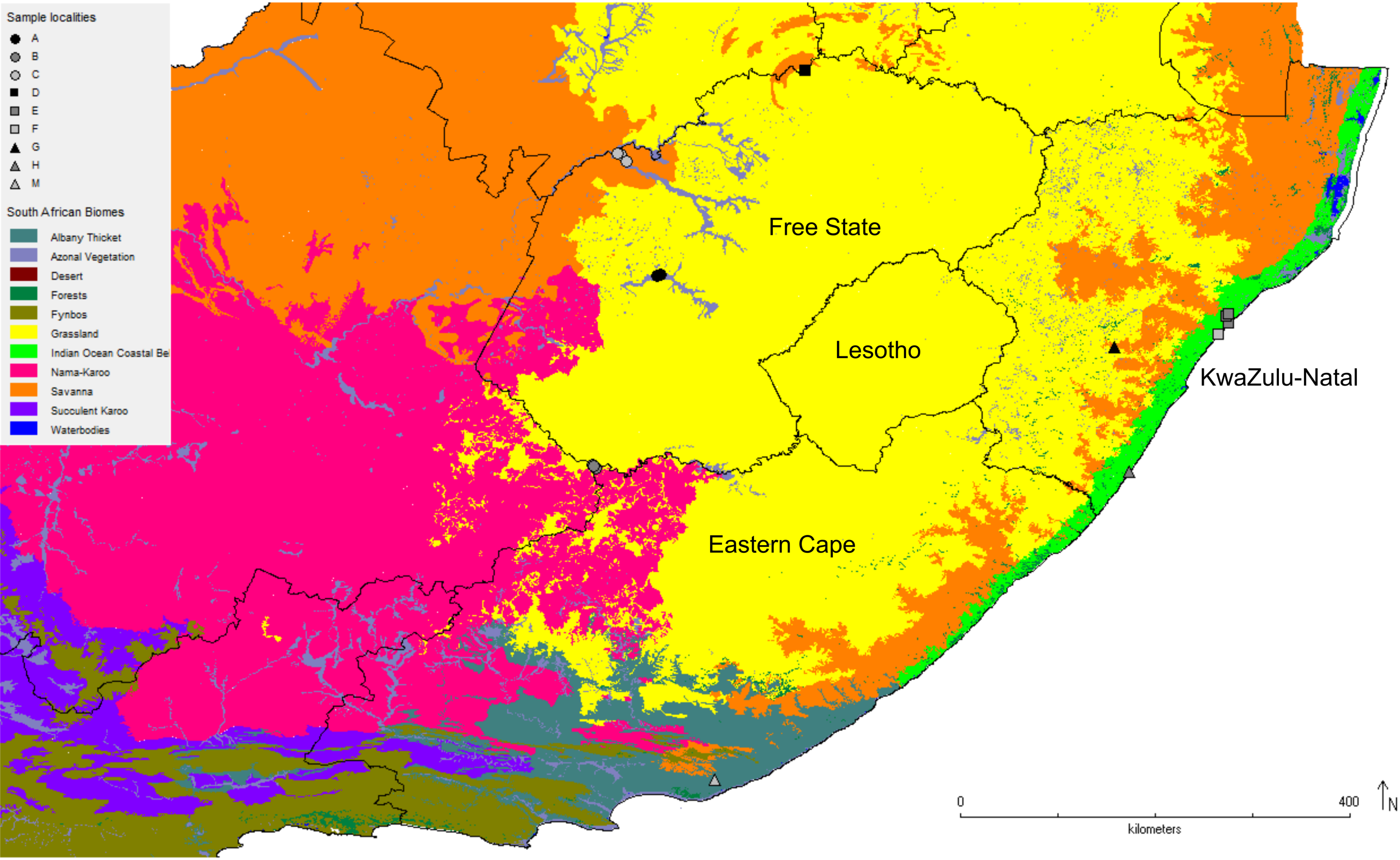

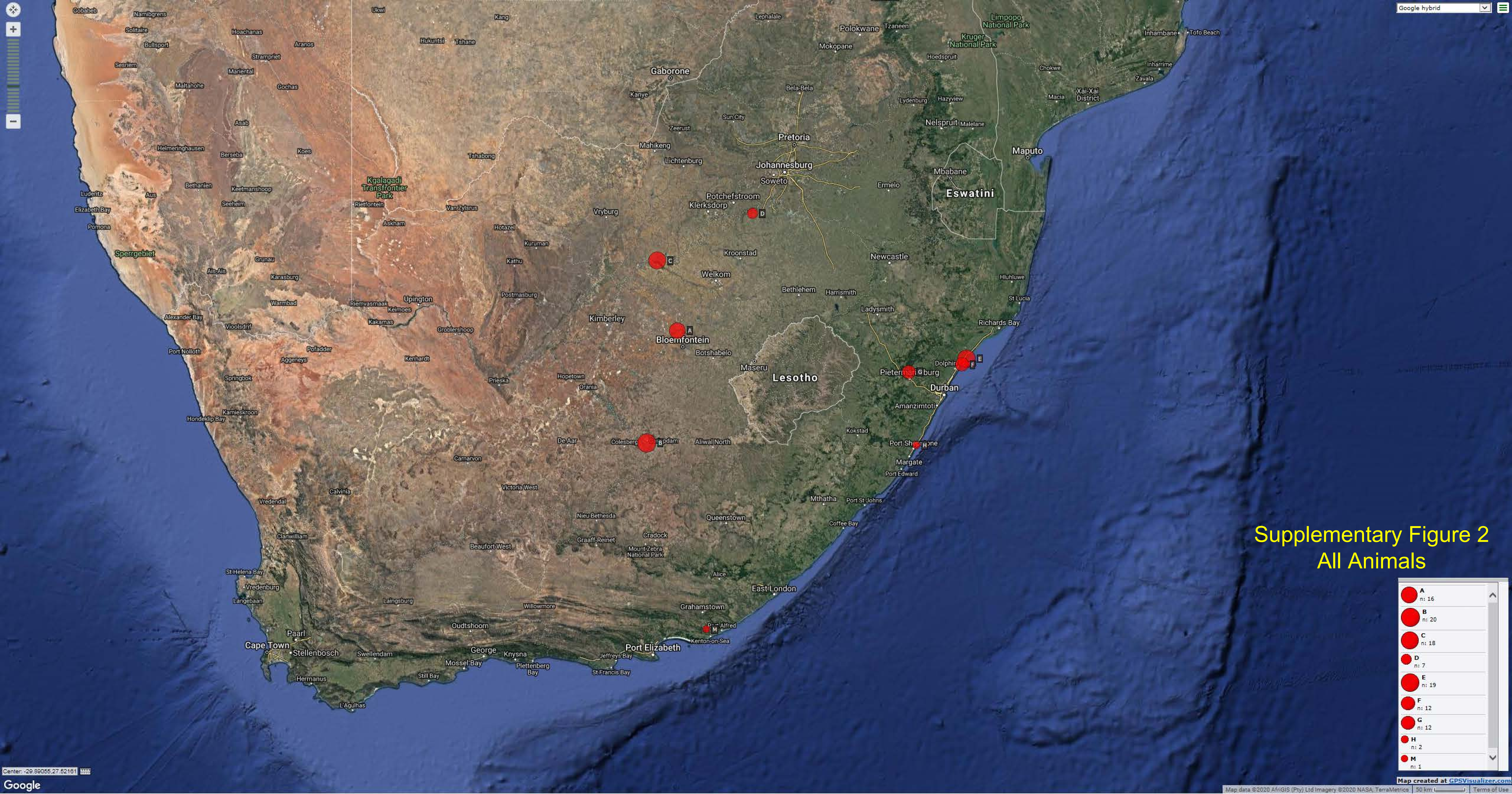

Supplementary Figure 2
All Animals

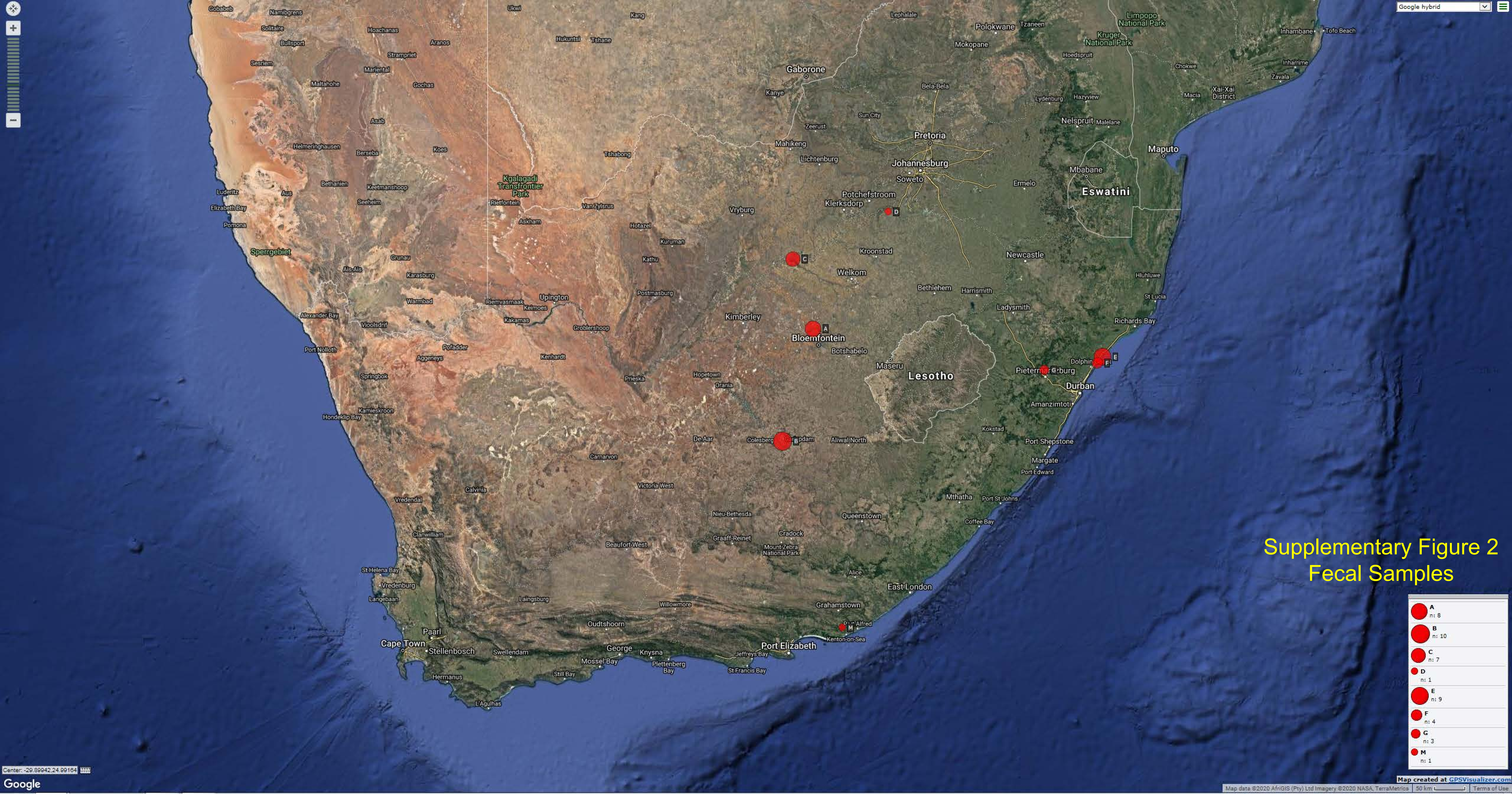

Supplementary Figure 2
Fecal Samples

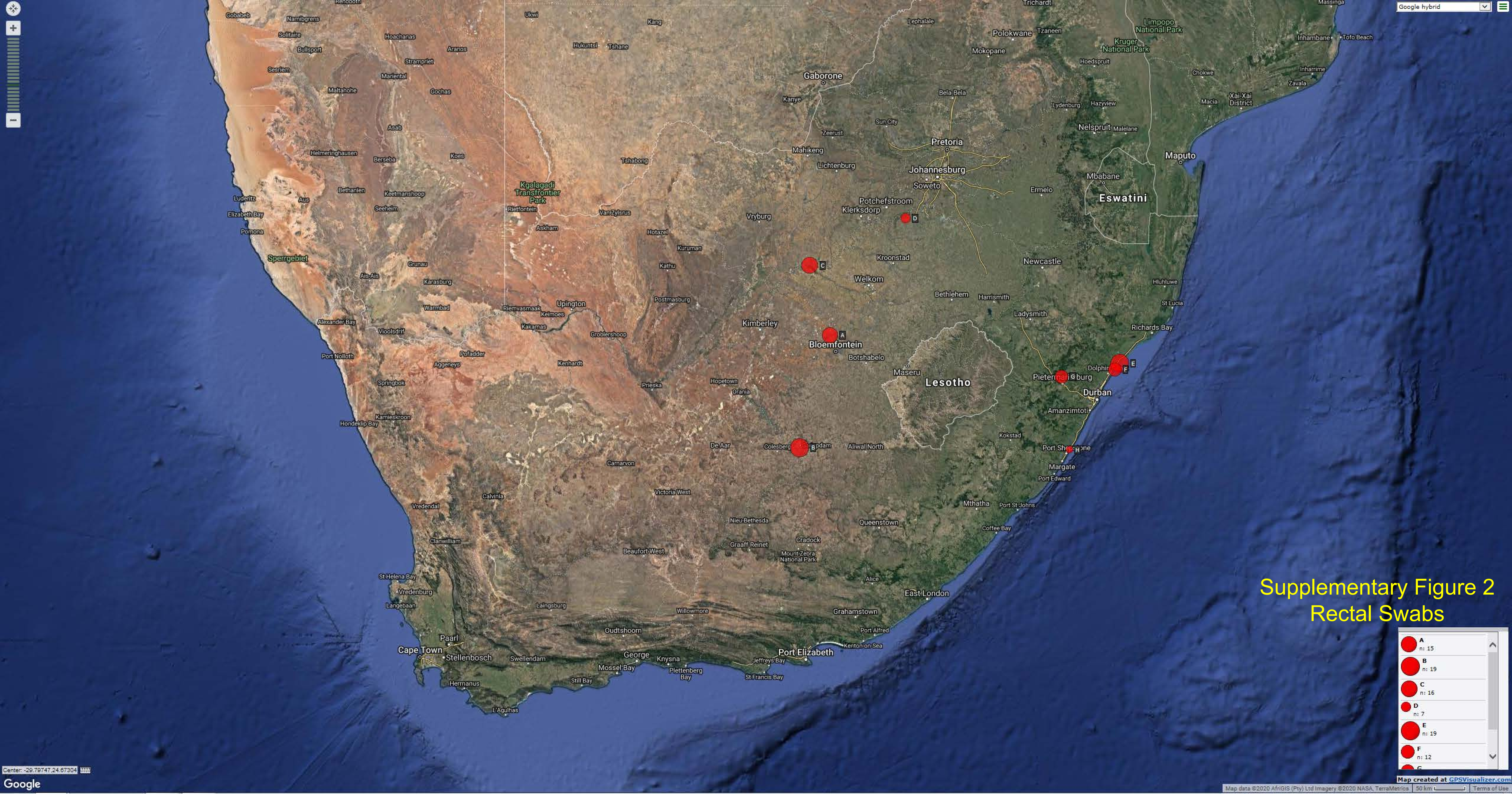

Supplementary Figure 2
Rectal Swabs

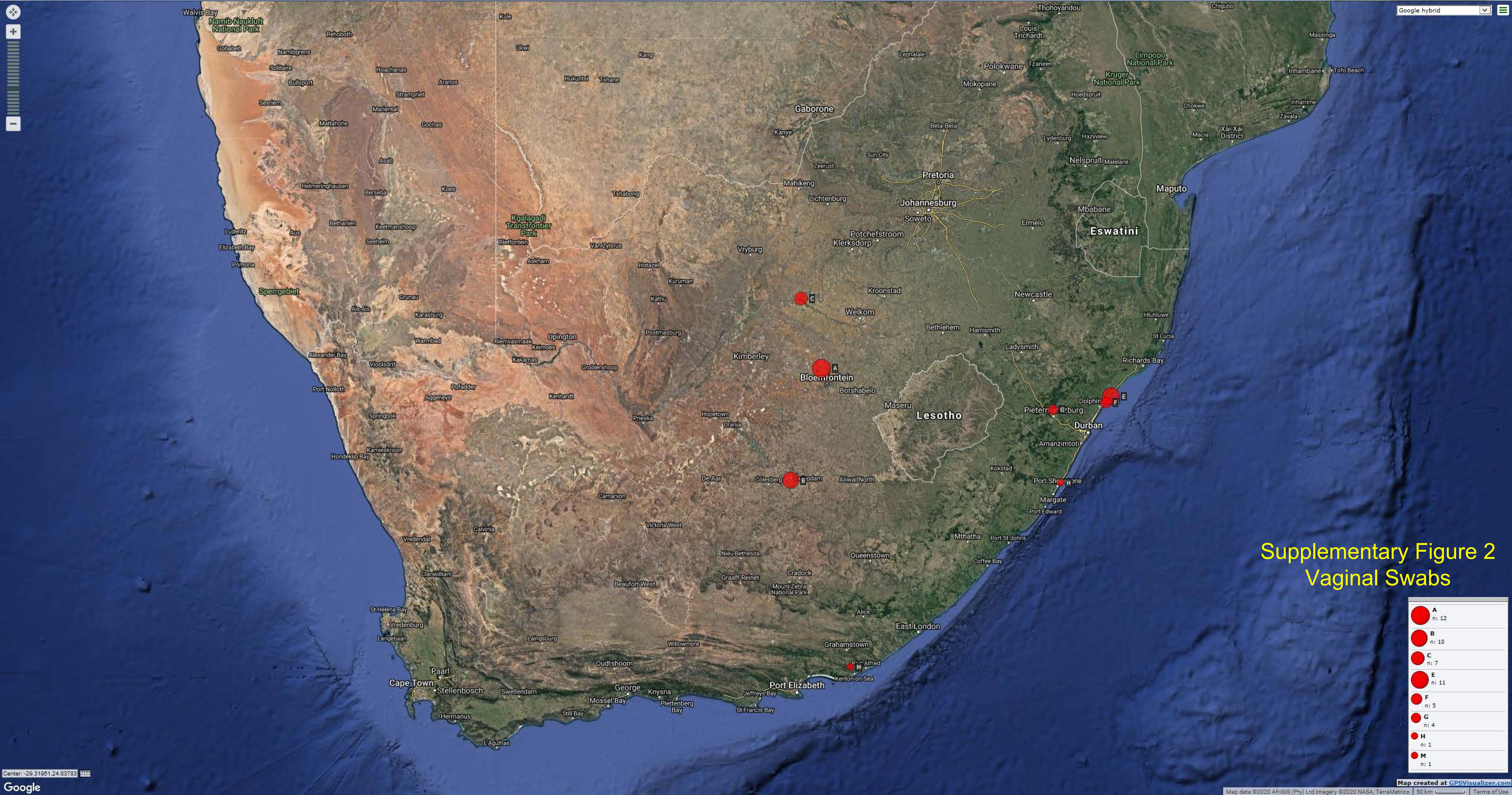

Supplementary Figure 2
Vaginal Swabs

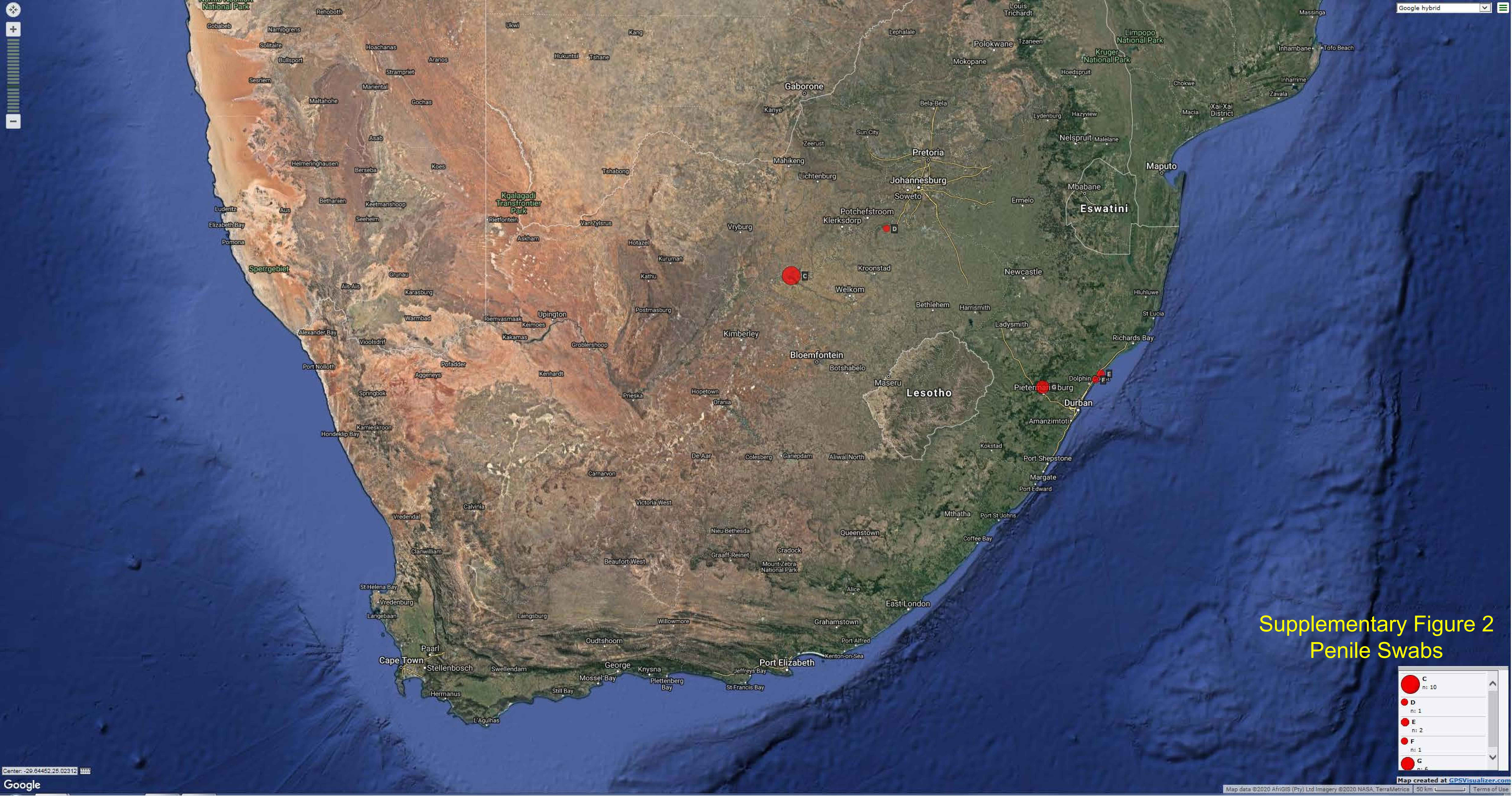

Supplementary Figure 2
Penile Swabs

Supplementary Figure 3

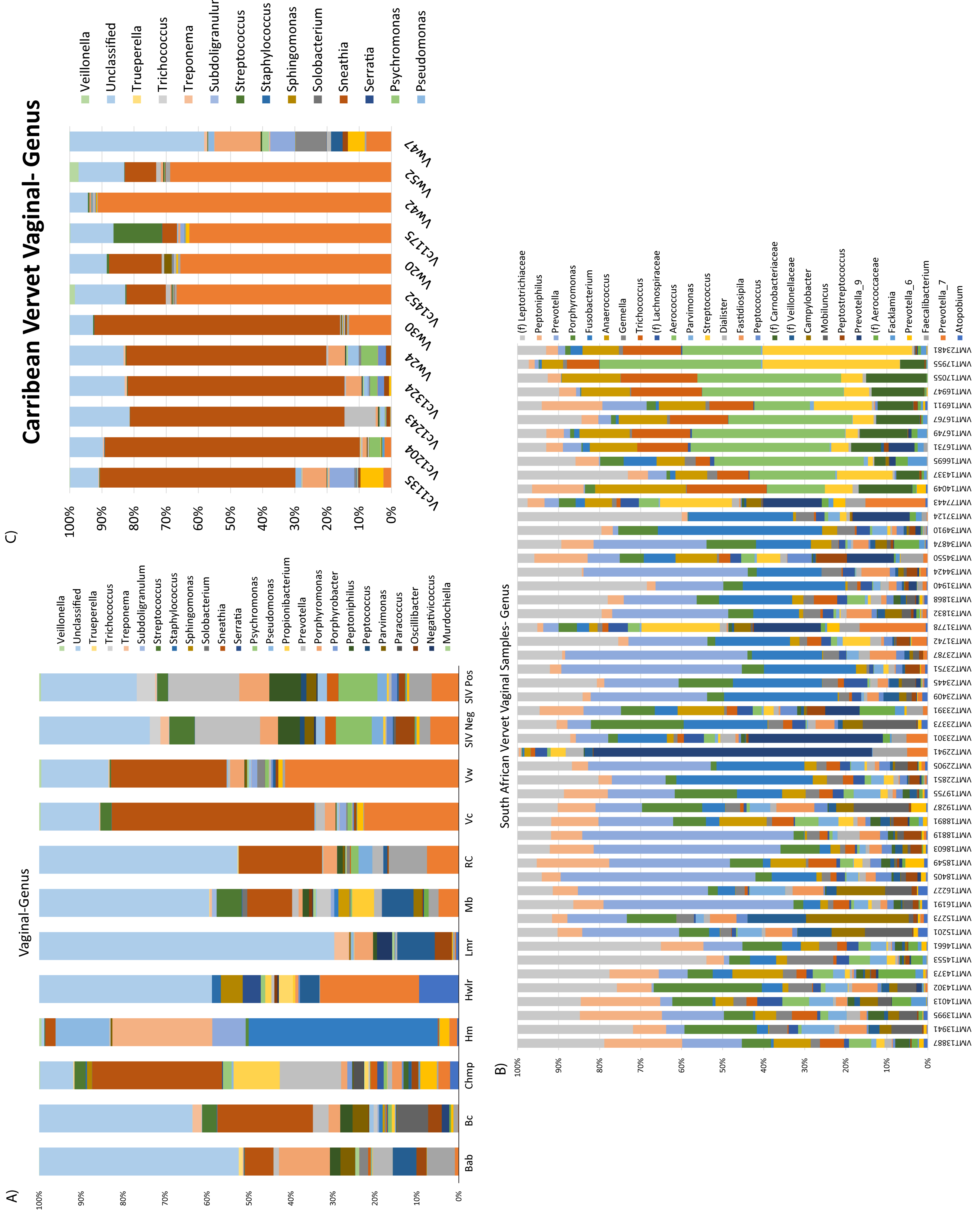

Supplementary Figure 4

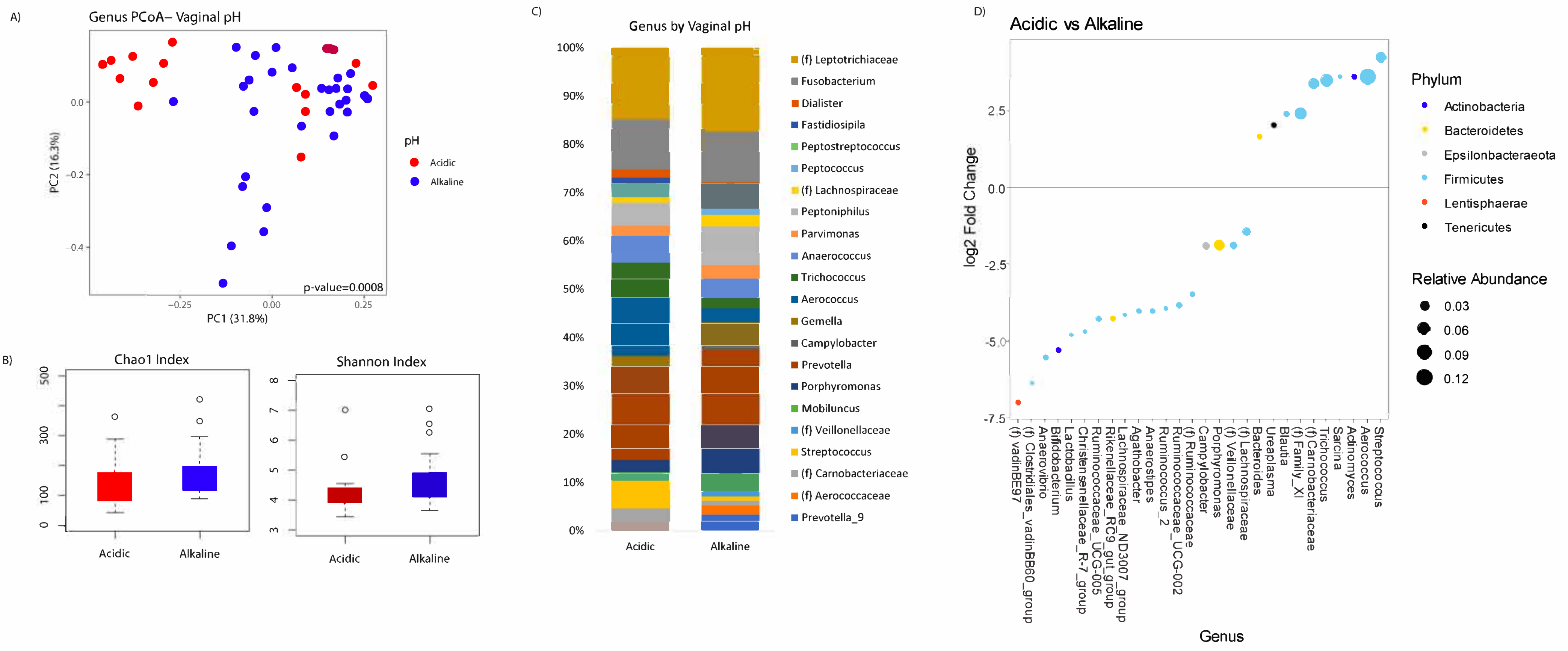

Supplementary Figure 5

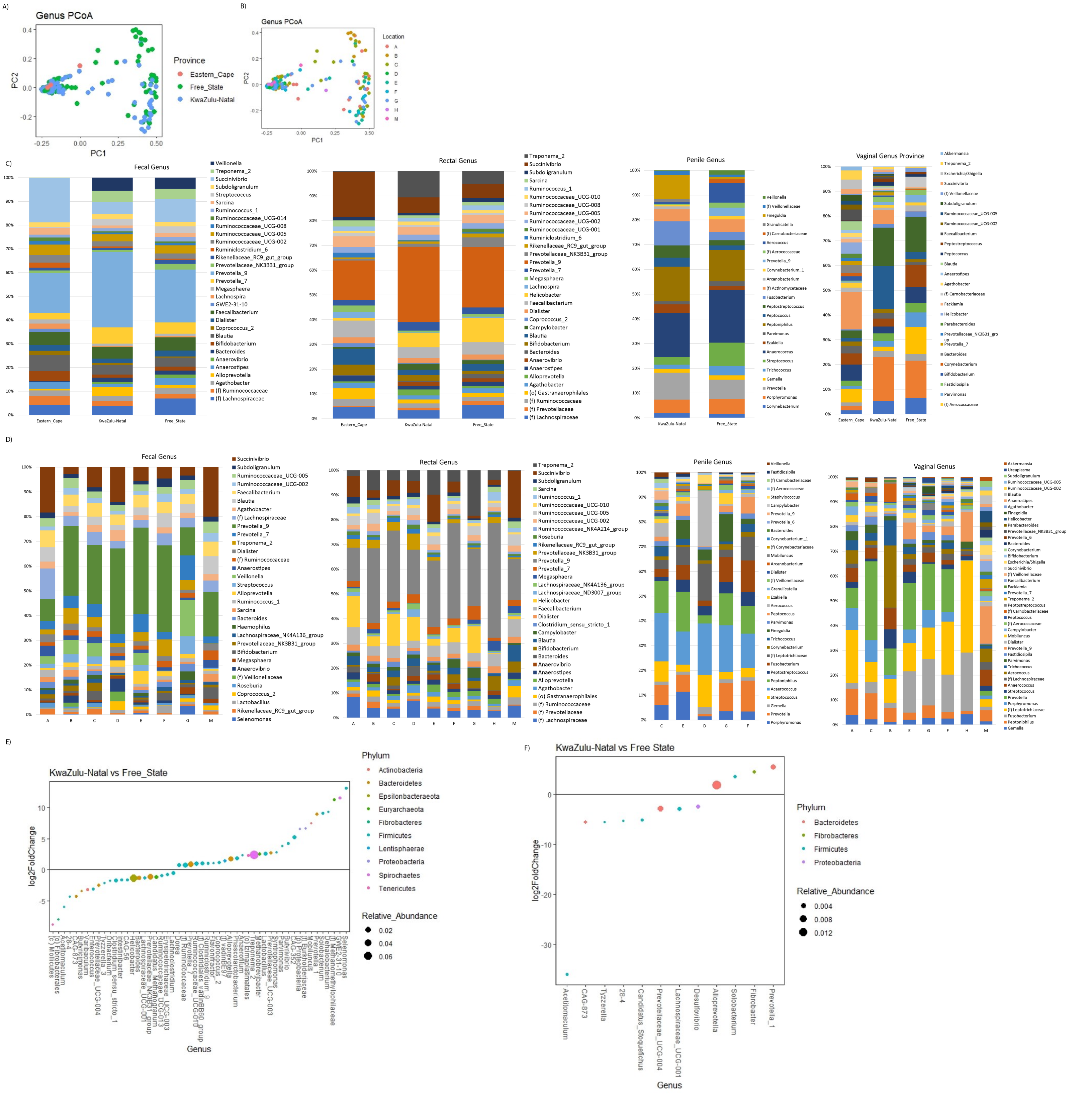

Supplementary Figure 6

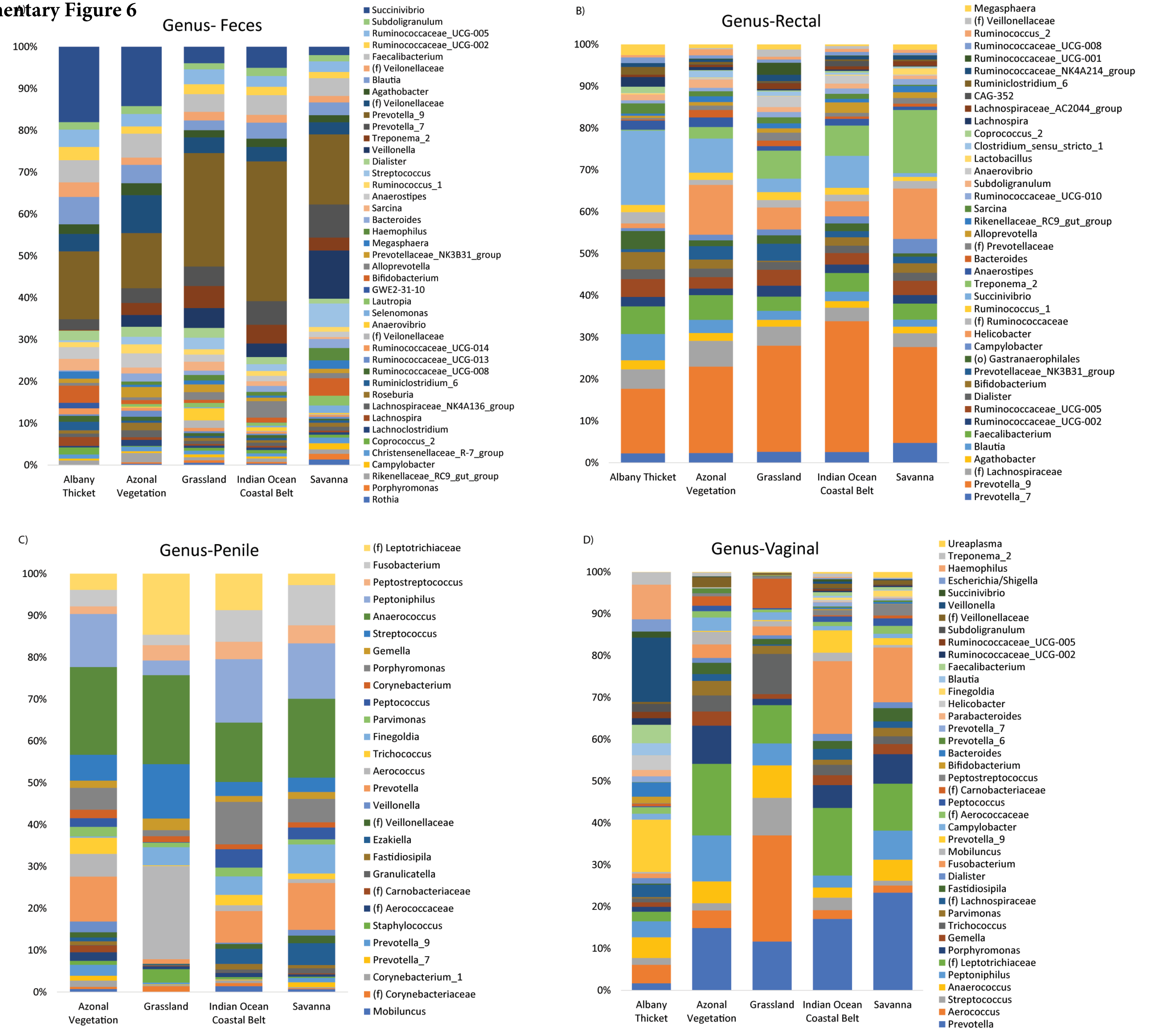

Supplementary Figure 7

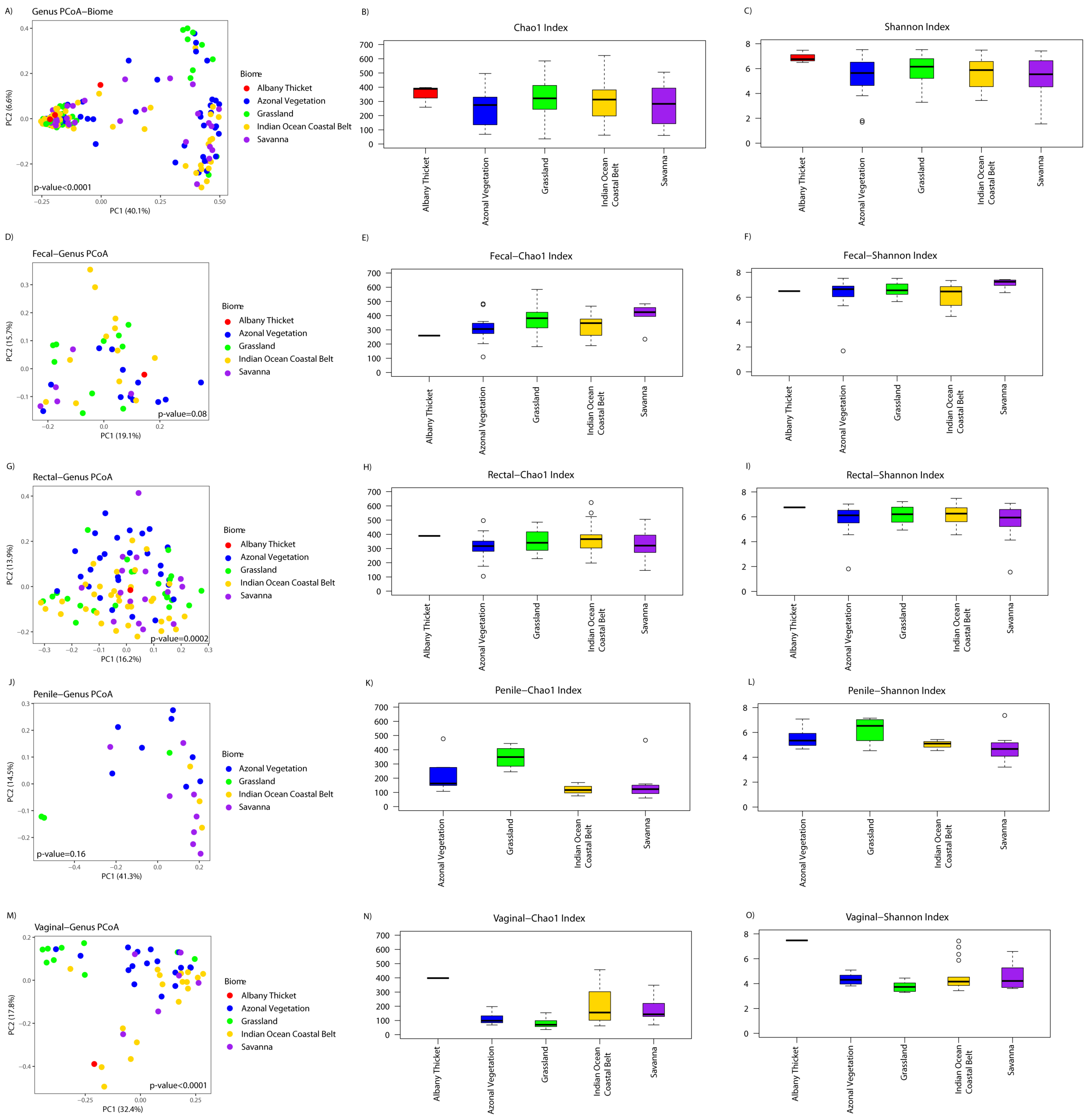

Supplementary Figure 8

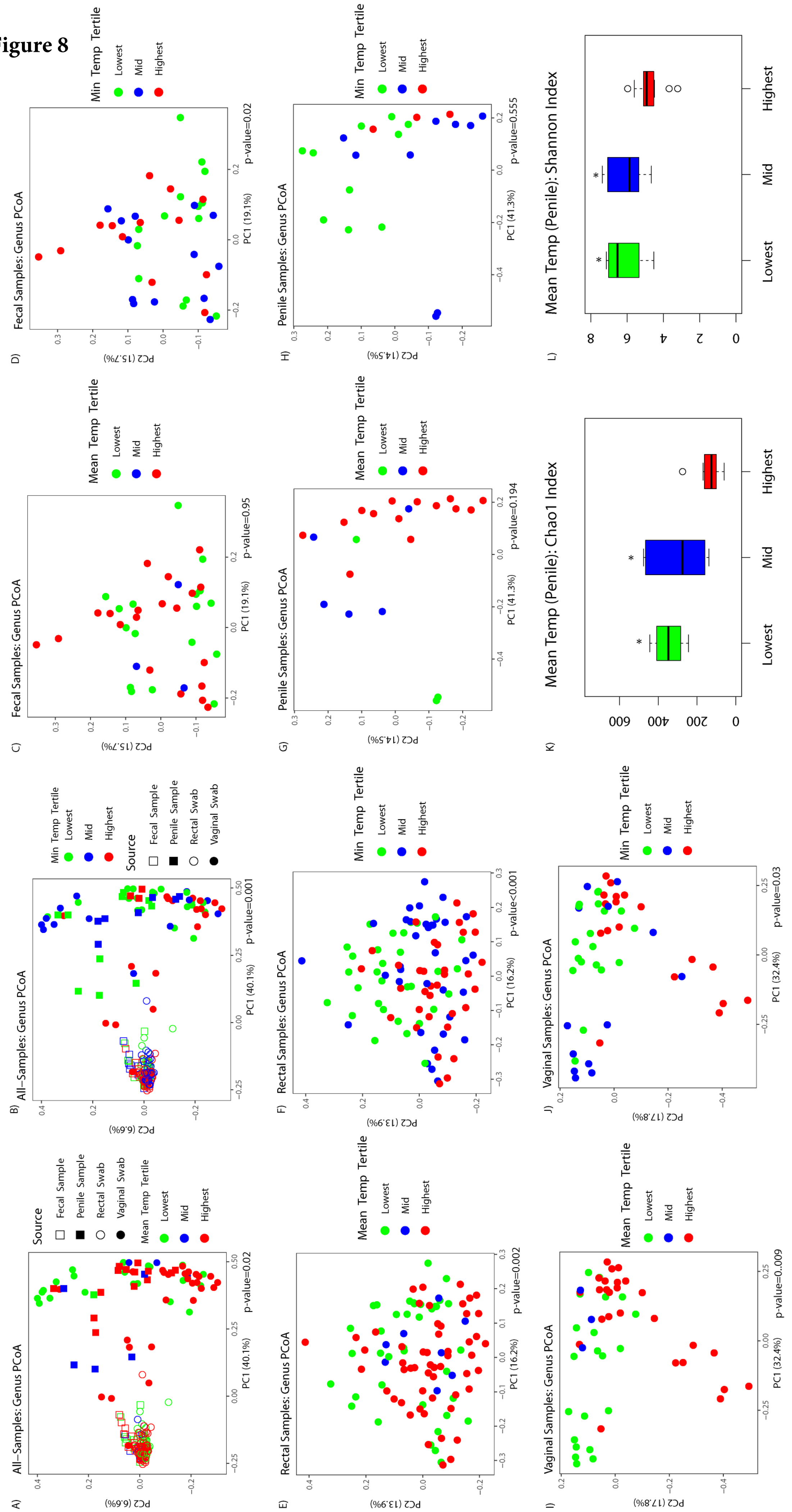

Supplementary Figure 9

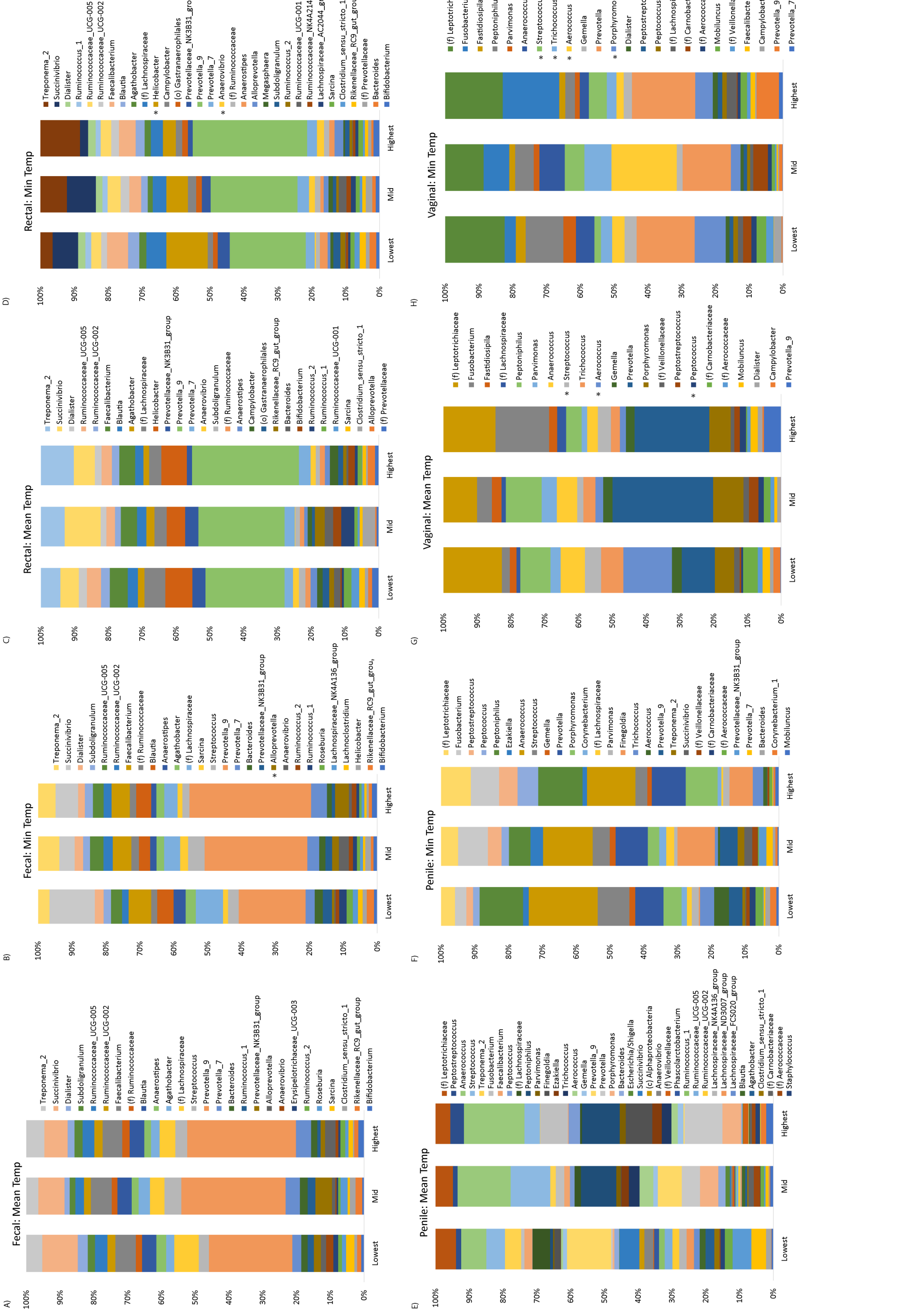

Supplementary Figure 10

B)

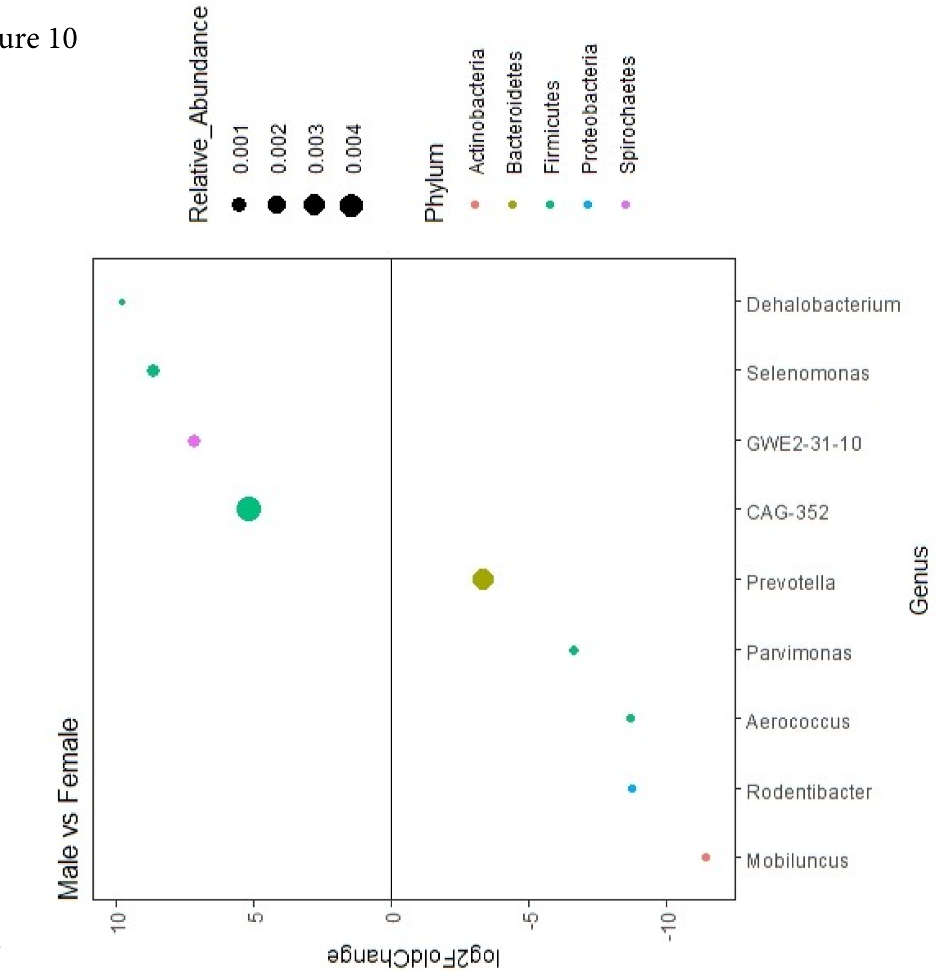

A)

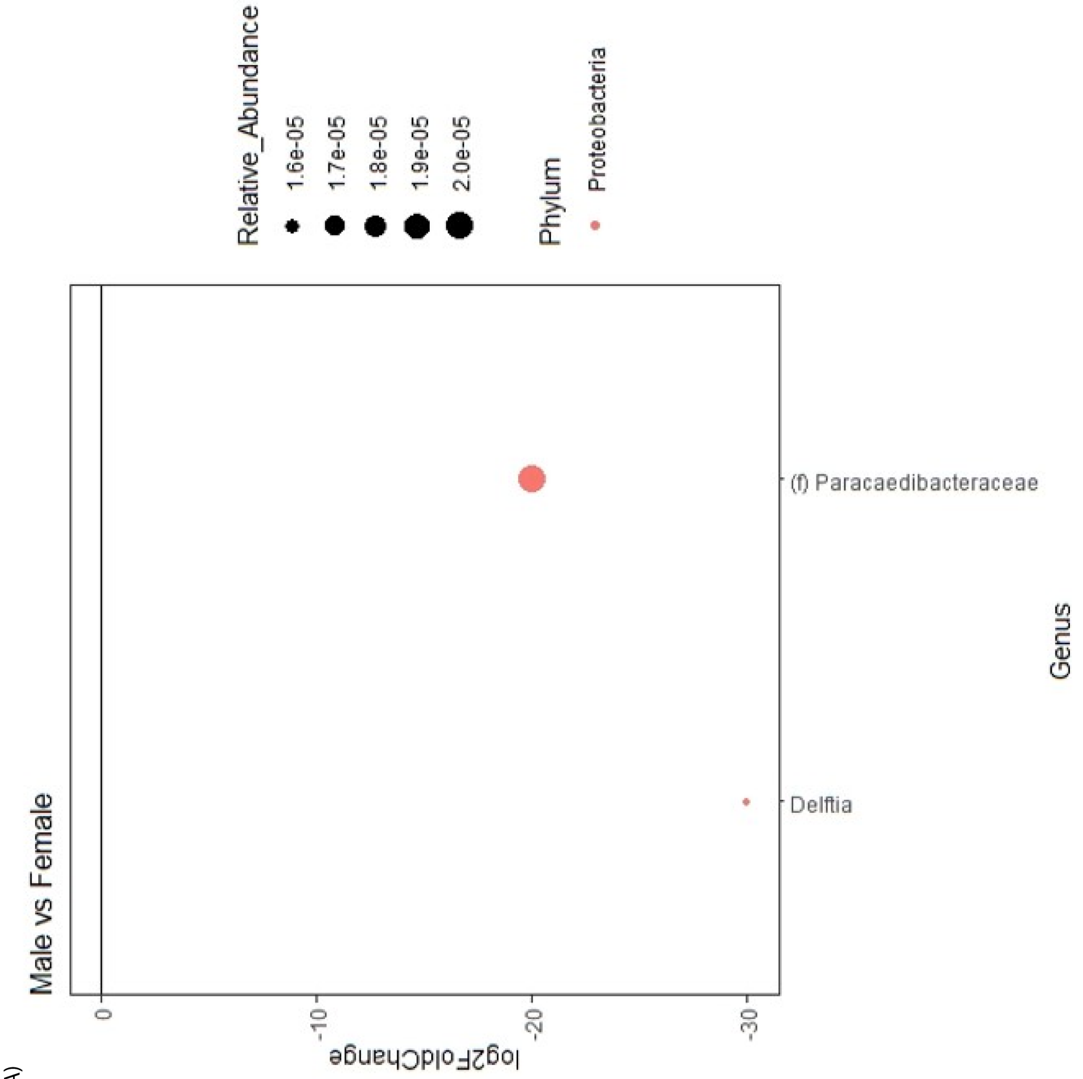

Supplementary Figure 11

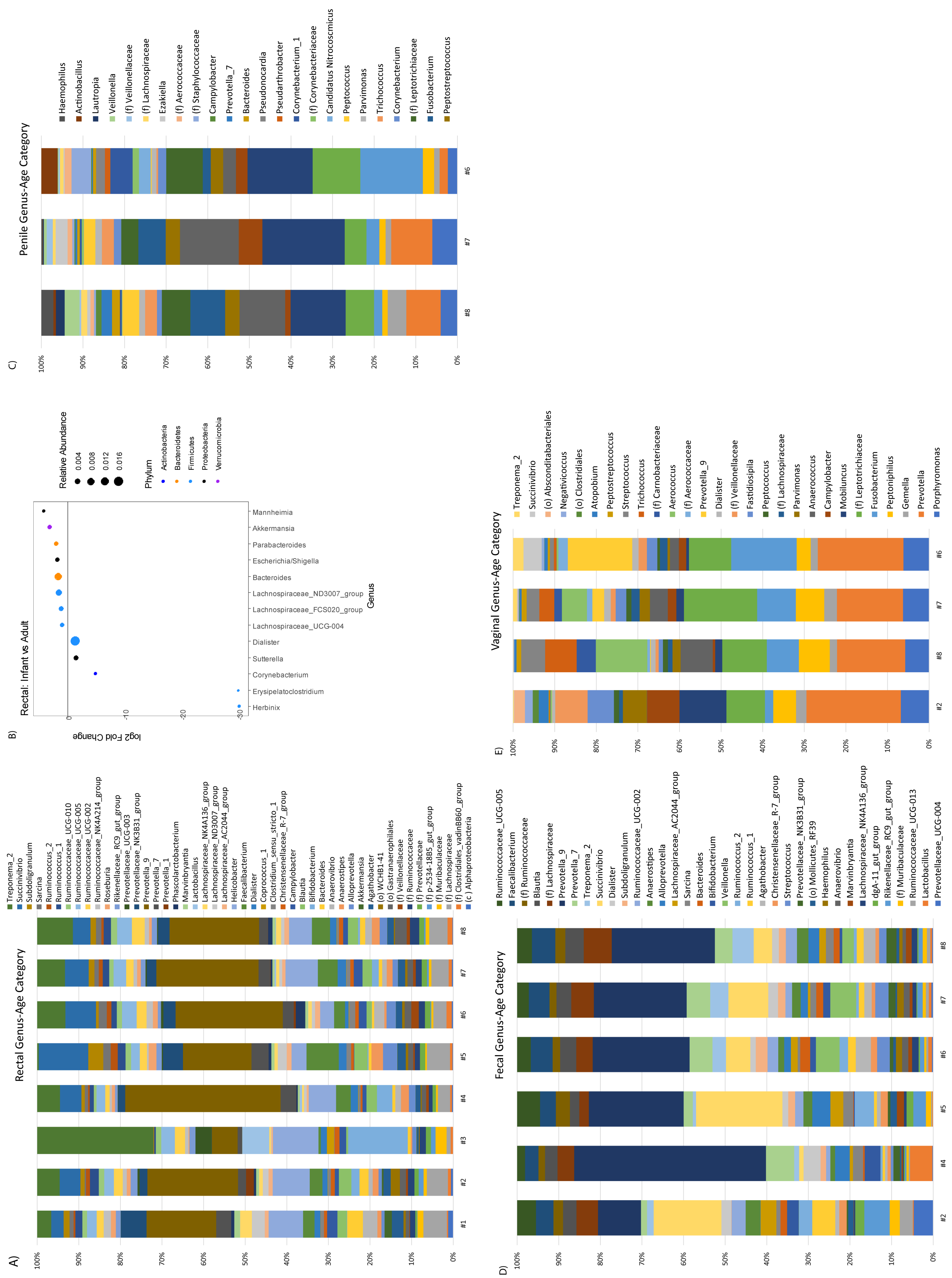

Supplementary Figure 12

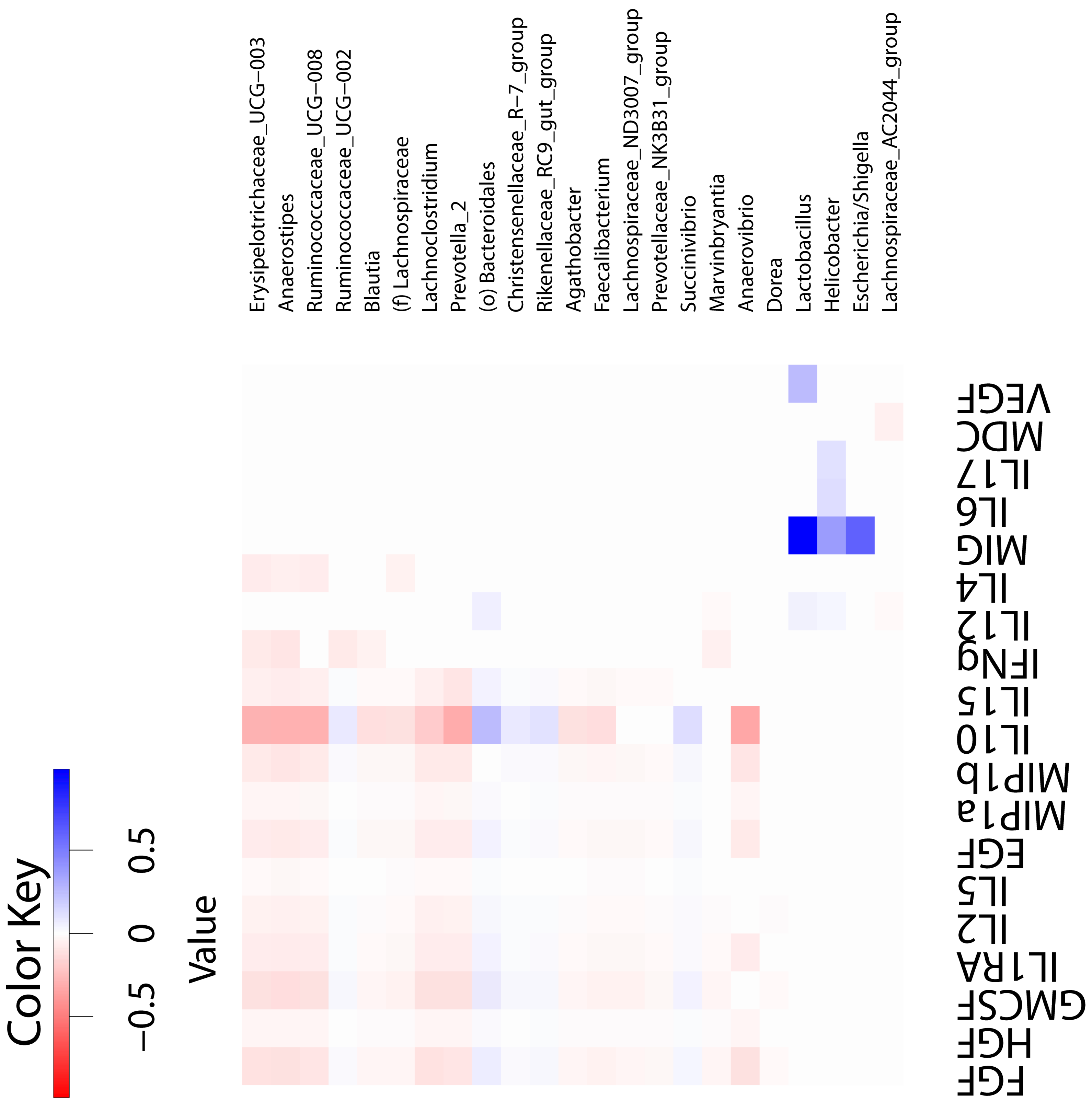

Supplementary Figure 13

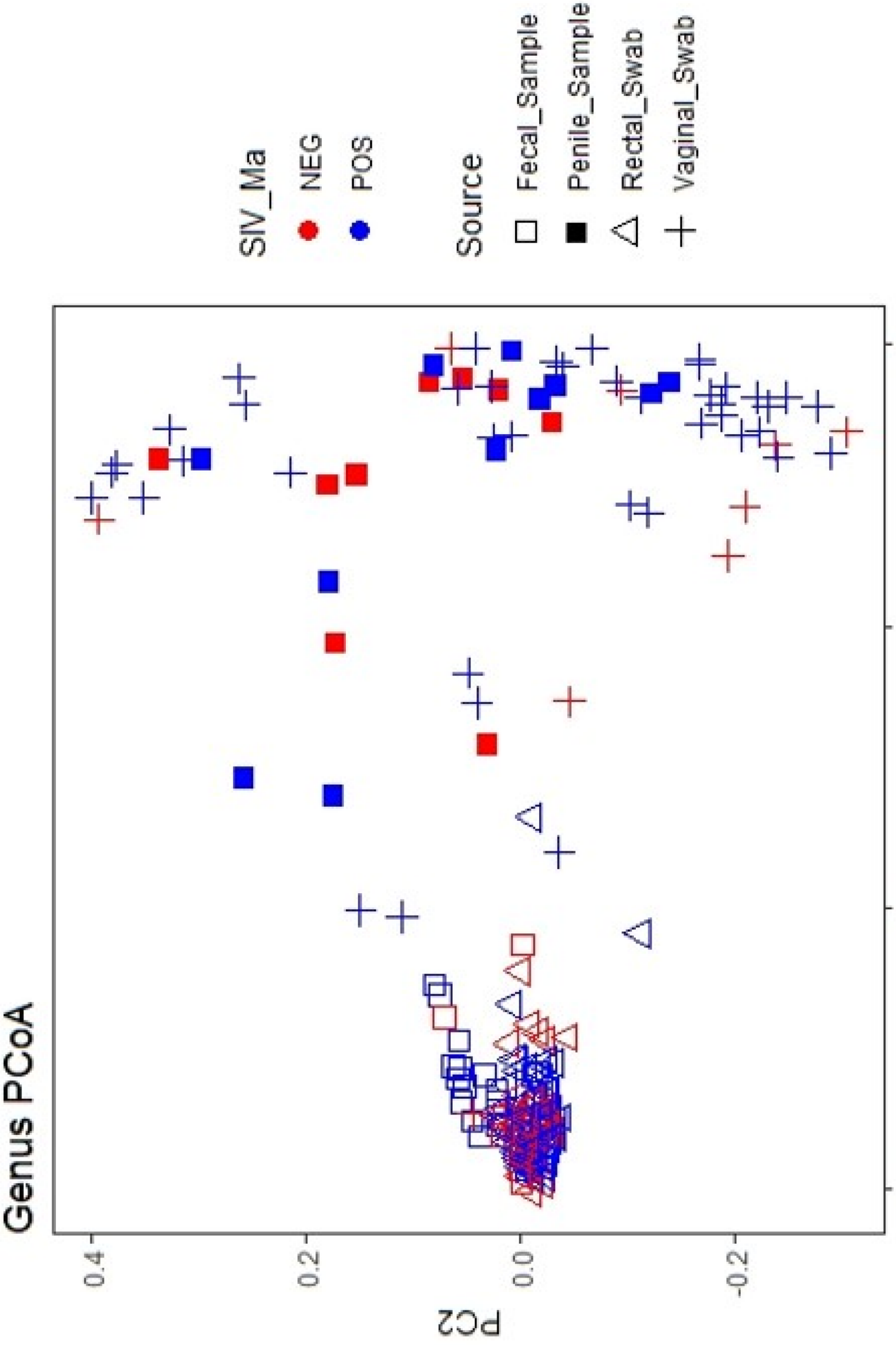

Supplementary Figure 14

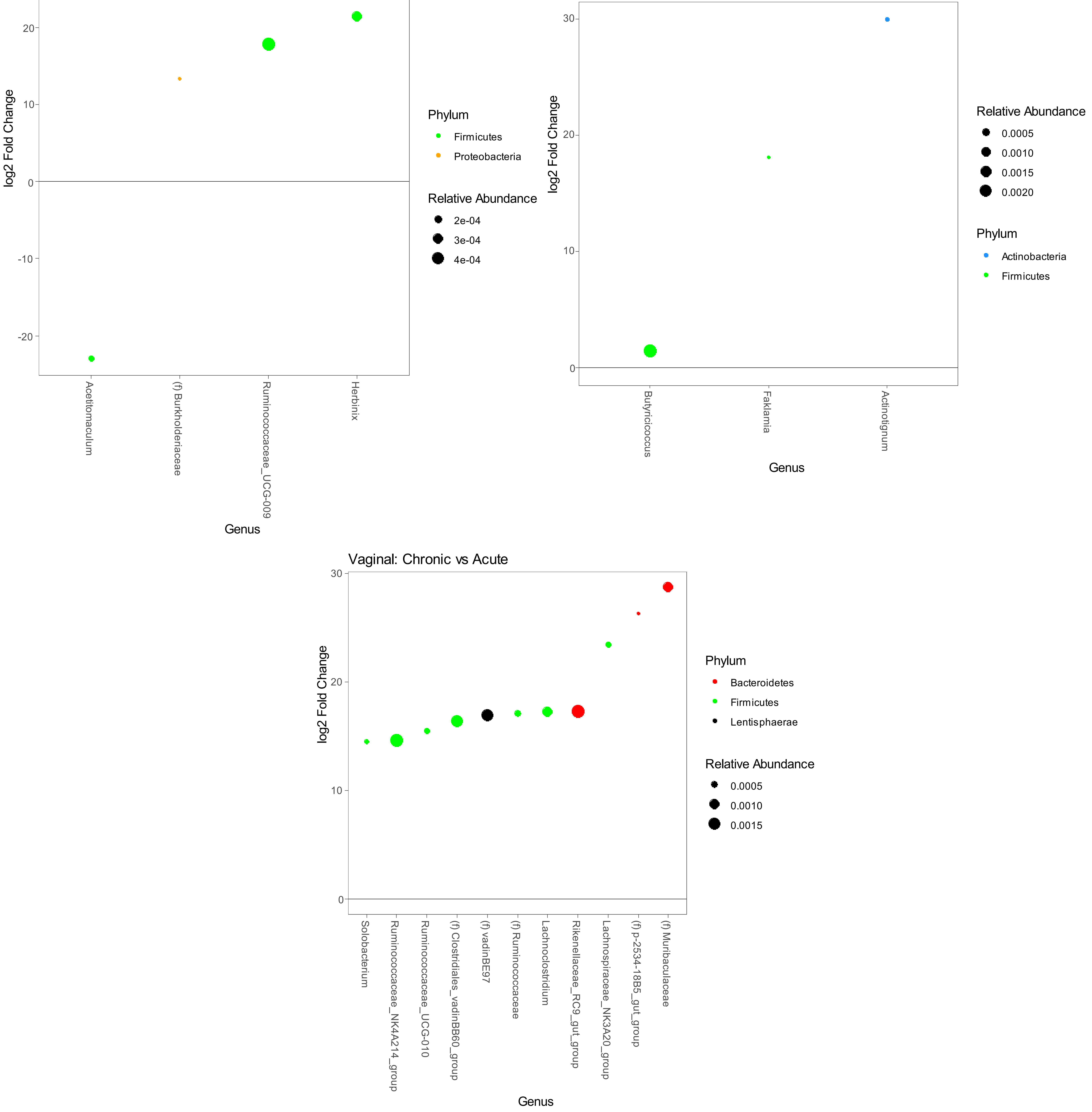

Supplementary Figure 15

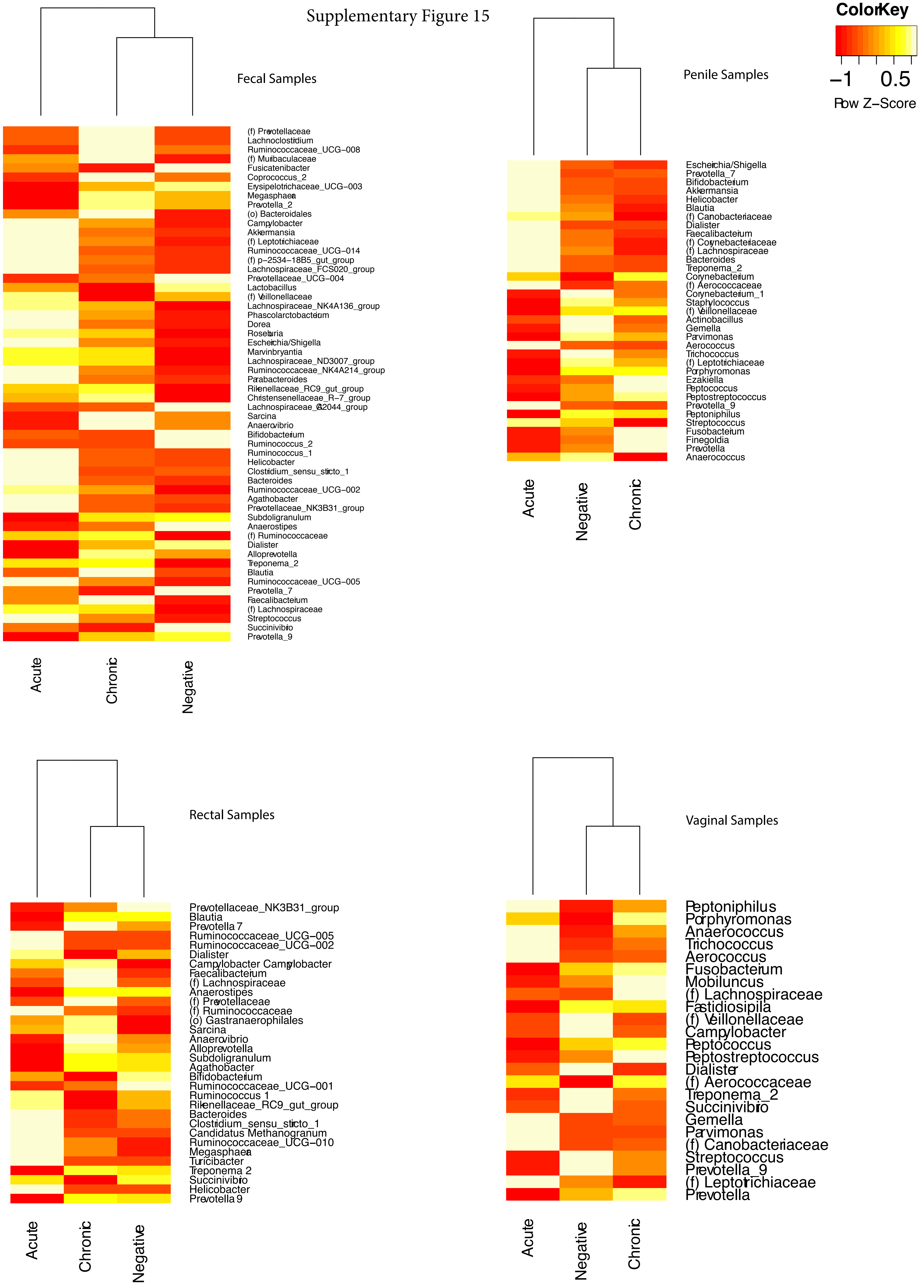

Supplementary Figure 16

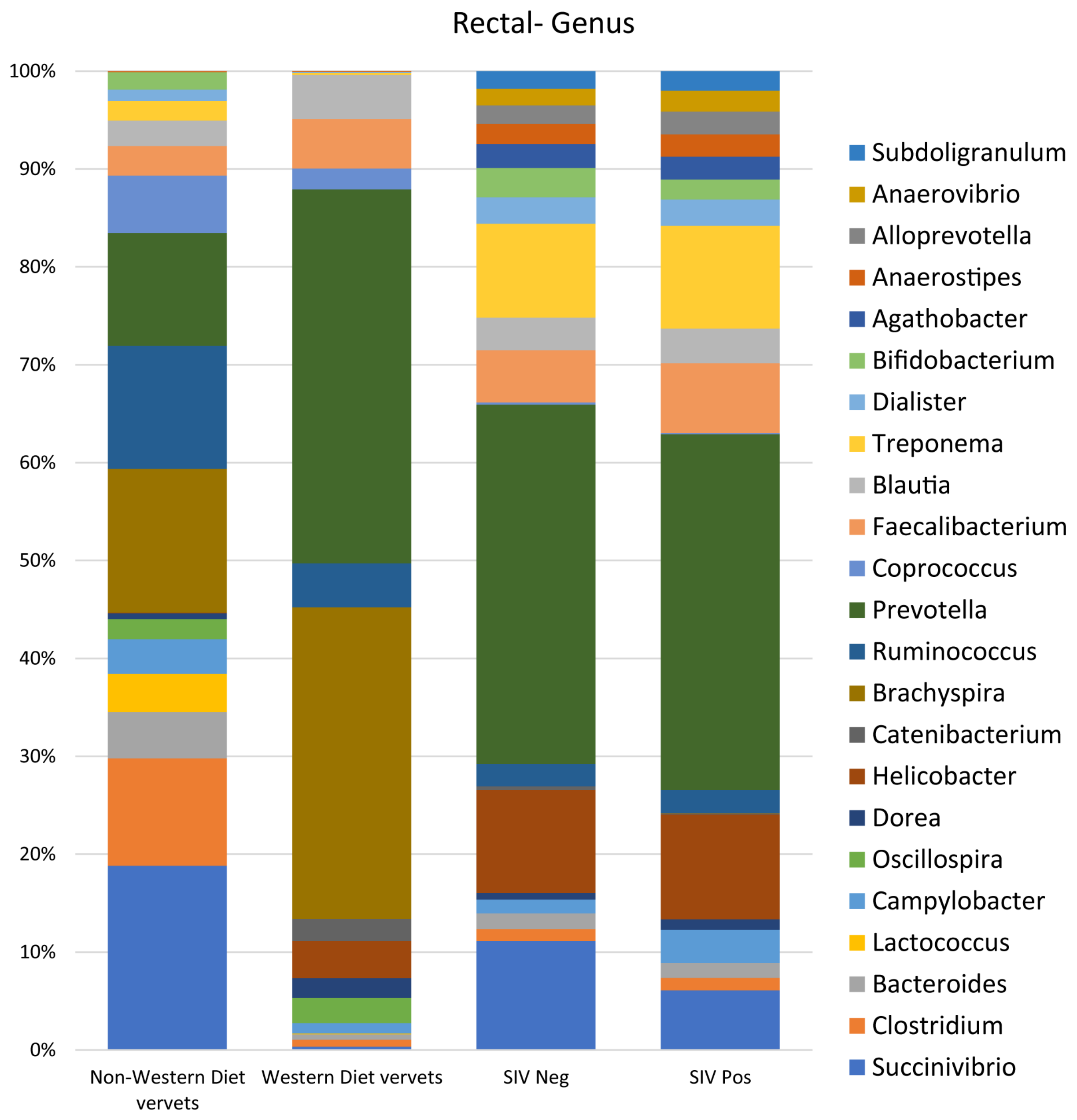

Supplement: Supplementary file 2 — Additional file 1 : Supplementary Figure 1. Geographic origins of study animals. Supplementary Figure 2. Maps of sampling sites. The maps were generated using GPS Visualizer [90] with Google Hybrid Map as a background using Map data © 2020 AfriGIS (Pty) Ltd Imagery© 2020 NASA, TerraMetrix. Supplementary Figure 3. Vaginal microbiome in South African vervets in our study compared to Caribbean-origin vervets and other NHPs based on the Yildrim et al. 2014 data [35]. (A) Average community structure in South African vervets (by SIV status), Caribbean origin vervets and other NHPs [35]. Species demarcation: African vervets (SIV Pos and SIV Neg), free-ranging Caribbean vervets (Vw), captive Caribbean-origin vervets (Vc), mangabeys (M), red colobus (Rc), yellow baboon (Bab), olive baboon (Bc), chimpanzee (Chimp), humans (H), lemurs (L), black howler (Bh). (B) Microbial community structure per individual in South African vervets, (C) Community structure per individual in Caribbean-origin vervets [35]. Supplementary Figure 4. A) PCoA of beta-diversity colored by vaginal pH. Acidic pH is a pH <7 while an alkaline pH is a pH > 7. B) Chao1 and Shannon Index of vaginal samples pH. C) Genus taxonomic plots of vaginal samples by pH. D) Differentially abundant microbial genera in acidic vaginal samples vs. alkaline vaginal samples. Supplementary Figure 5. Geography-related microbiome. (A) PCoA for each body site clustering by province. (B) PCoA for each body site clustering by geographic site. (C) Community structure by province. (D) Community structure by geographic site. Differentially abundant taxa between KZN and FS in (E) rectal and (F) fecal microbiome. Supplementary 6. Genus taxonomic plots of D) fecal, E) rectal, F) penile, and G) vaginal samples by the different biomes. Supplementary Figure 7. PCoA of beta-diversity, Chao1 index and Shannon index by different biomes for (A-C) all samples, (D-F) fecal samples, (G-I) rectal samples, (J-L) penile samples, and (M-O) vaginal sam [file 40168_2020_928_MOESM1_ESM.pdf]
