# Supplementary material for: Shifts in microbial diversity, composition, and functionality in the gut and genital microbiome during a natural SIV infection in vervet monkeys
Source: Microbiome. 2020 Nov 6;8:154. doi: 10.1186/s40168-020-00928-4 (PMC7648414; doi:10.1186/s40168-020-00928-4)
Supplement: Supplementary file 3 — Additional file 2 : Supplementary Table 1. Characteristics of microbial samples and individuals used in the studies. Supplementary Table 2. Relative abundances at the phylum and at the genus level within the bacterial communities in four sample type. Showing taxa with > 1% relative abundance in at least one sample type. Supplementary Table 3. Relative abundances of predicted metagenome categorized by function across fecal enterotypes. Only showing genes with significant differential abundance between the enterotypes and at a relative abundance > 0.1%. Data is expressed as relative abundance (%). Supplementary Table 4. Vaginal pH. [file 40168_2020_928_MOESM2_ESM.docx]

**Supplementary Table 1. Characteristics of microbial samples and individuals used in the studies.**

| **Samples** | **Fecal samples**  **N=44** | **Rectal swabs**  **N=103** | **Penile swabs**  **N=20** | **Vaginal swabs**  **N=51** |
| --- | --- | --- | --- | --- |
| **by province** |  |  |  |  |
| KwaZulu-Natal (KZN) | 16 | 45 | 9 | 21 |
| Free State (FS) | 27 | 57 | 11 | 29 |
| Eastern Cape (EC) | 1 | 1 | 0 | 1 |
| **by geographic site** |  |  |  |  |
| A (FS) | 8 | 15 | 0 | 12 |
| B (FS) | 11 | 19 | 0 | 10 |
| C (FS) | 7 | 16 | 10 | 7 |
| D (FS) | 1 | 7 | 1 | 0 |
| E (KZN) | 9 | 19 | 2 | 11 |
| F (KZN) | 4 | 12 | 1 | 5 |
| G (KZN) | 3 | 12 | 6 | 4 |
| H (KZN) | 0 | 2 | 0 | 1 |
| M (EC) | 1 | 1 | 0 | 1 |
| **by developmental stage** |  |  |  |  |
| infant | 1 | 10 | 0 | 1 |
| young juvenile | 2 | 9 | 0 | 0 |
| older juvenile | 7 | 8 | 2 | 3 |
| adult | 34 | 76 | 18 | 47 |
| **by age category** |  |  |  |  |
| 1 | 0 | 3 | 0 | 0 |
| 2 | 1 | 7 | 0 | 1 |
| 3 | 0 | 1 | 0 | 0 |
| 4 | 2 | 8 | 0 | 0 |
| 5 | 1 | 2 | 0 | 0 |
| 6 | 6 | 6 | 2 | 3 |
| 7 | 22 | 60 | 16 | 34 |
| 8 | 12 | 16 | 2 | 13 |
| **by SIV infection status*** |  |  |  |  |
| SIV positive | 32 | 62 | 11 | 41 |
| SIV negative | 11 | 41 | 9 | 10 |
| **by stage of SIV infection**** |  |  |  |  |
| acute infection | 4 | 11 | 2 | 6 |
| chronic infection | 23 | 43 | 8 | 30 |

* SIV-negative or SIV-positive (for pol, env, and/or gag)

** based on the VL threshold of 10^6 RNA copies/ml

**Supplementary Table 2. Relative abundances at the phylum and at the genus level within the bacterial communities in four sample type. Showing taxa with > 1% relative abundance in at least one sample type.**

Phylum

| Phylum | Vaginal_Swab | Penile_Sample | Fecal_Sample | Rectal_Swab |
| --- | --- | --- | --- | --- |
| Actinobacteria | 0.034899038 | 0.042404398 | 0.010737499 | 0.012680475 |
| Bacteroidetes | 0.249342231 | 0.189655497 | 0.337841234 | 0.329906404 |
| Cyanobacteria | 0.000646503 | 0.001891506 | 0.004707947 | 0.012235029 |
| Epsilonbacteraeota | 0.012436086 | 0.007253285 | 0.011946442 | 0.071098015 |
| Firmicutes | 0.458410608 | 0.62203416 | 0.492824335 | 0.41313632 |
| Fusobacteria | 0.219145507 | 0.092921171 | 0.004348297 | 0.002053904 |
| Proteobacteria | 0.007950429 | 0.021010034 | 0.073149753 | 0.057469451 |
| Spirochaetes | 0.006430367 | 0.009845627 | 0.036014819 | 0.060099662 |

Genus

| Genus | Vaginal_Swab | Penile_Sample | Fecal_Sample | Rectal_Swab |
| --- | --- | --- | --- | --- |
| (f) Aerococcaceae | 0.010687235 | 0.00967607 | 0.000298986 | 4.07E-05 |
| (f) Carnobacteriaceae | 0.019651616 | 0.006111599 | 0 | 4.94E-05 |
| (f) Lachnospiraceae | 0.016484964 | 0.01346285 | 0.041468065 | 0.033838288 |
| (f) Leptotrichiaceae | 0.139450559 | 0.041549076 | 0.002404888 | 0.000904758 |
| (f) Prevotellaceae | 0.001114481 | 0.000572727 | 0.006848514 | 0.010306786 |
| (f) Ruminococcaceae | 0.001409134 | 0.002596111 | 0.014431493 | 0.011002687 |
| (f) Veillonellaceae | 0.011860647 | 0.009762733 | 0.003039691 | 0.003284513 |
| (o) Gastranaerophilales | 0.000587573 | 0.001593839 | 0.003949649 | 0.011844181 |
| Aerococcus | 0.06549441 | 0.018688983 | 0.00093379 | 0.000131727 |
| Agathobacter | 0.001402201 | 0.005022664 | 0.018528469 | 0.013554901 |
| Alloprevotella | 0.001662189 | 0.001533552 | 0.016472398 | 0.012263628 |
| Anaerococcus | 0.045194558 | 0.138038486 | 0.001085449 | 0.00099922 |
| Anaerostipes | 0.001237542 | 0.00290885 | 0.017332524 | 0.012040905 |
| Anaerovibrio | 0.000748765 | 0.004144734 | 0.009825375 | 0.010898691 |
| Bacteroides | 0.002622411 | 0.007927746 | 0.013736026 | 0.008905451 |
| Bifidobacterium | 0.001242742 | 0.001744556 | 0.009151573 | 0.010476644 |
| Blautia | 0.002549614 | 0.005147006 | 0.031020886 | 0.019818875 |
| Campylobacter | 0.010170725 | 0.002211781 | 0.003282347 | 0.015259555 |
| Corynebacterium | 0.001982841 | 0.014046881 | 0.000119161 | 0.000281654 |
| Dialister | 0.010538175 | 0.006360283 | 0.017410521 | 0.015841927 |
| Ezakiella | 0.001017419 | 0.019792989 | 9.53E-05 | 0.000597105 |
| Faecalibacterium | 0.005340151 | 0.0072985 | 0.043214317 | 0.036640957 |
| Fastidiosipila | 0.019752145 | 0.005516264 | 0.000415981 | 0.000104862 |
| Finegoldia | 0.003059191 | 0.046624491 | 2.82E-05 | 0.000103995 |
| Fusobacterium | 0.079694948 | 0.051372095 | 0.001943409 | 0.001149146 |
| Gemella | 0.023929283 | 0.013511833 | 0.001442933 | 0.000103129 |
| Helicobacter | 0.002265361 | 0.005041504 | 0.008664096 | 0.055838461 |
| Mobiluncus | 0.018096889 | 0.005576551 | 0.000797296 | 9.97E-05 |
| Parvimonas | 0.021230609 | 0.012777085 | 0.000478811 | 0.000188925 |
| Peptococcus | 0.010976688 | 0.021627976 | 3.47E-05 | 0.000140393 |
| Peptoniphilus | 0.060920357 | 0.082359635 | 0.001410434 | 0.000699367 |
| Peptostreptococcus | 0.010314585 | 0.027121633 | 0.000121328 | 0.000365716 |
| Porphyromonas | 0.05463385 | 0.041537772 | 0.001871913 | 0.001249675 |
| Prevotella | 0.150333651 | 0.073180933 | 0.00208207 | 0.003128521 |
| Prevotella_7 | 0.004676315 | 0.008496705 | 0.026670422 | 0.022852067 |
| Prevotella_9 | 0.023034925 | 0.031511283 | 0.209270734 | 0.20836381 |
| Prevotellaceae_NK3B31 | 0.001372736 | 0.008089767 | 0.014011179 | 0.019192304 |
| Ruminococcaceae_UCG-002 | 0.001880579 | 0.003862139 | 0.017202531 | 0.016936476 |
| Ruminococcaceae_UCG-005 | 0.002007106 | 0.004363275 | 0.025394315 | 0.02536615 |
| Ruminococcus_1 | 0.001048618 | 0.003044496 | 0.01049051 | 0.011947309 |
| Sarcina | 0.000731433 | 0.001725717 | 0.012665742 | 0.009490424 |
| Streptococcus | 0.036552561 | 0.054243266 | 0.024755178 | 0.001272207 |
| Subdoligranulum | 0.0014386 | 0.003210285 | 0.016099749 | 0.011051218 |
| Succinivibrio | 0.003027992 | 0.008613511 | 0.061452466 | 0.046673889 |
| Treponema_2 | 0.006414767 | 0.009830556 | 0.035640003 | 0.059300633 |
| Trichococcus | 0.03920617 | 0.021624208 | 0.000626137 | 0.000100529 |

**Supplementary Table 3: Relative abundances of predicted metagenome categorized by function across fecal enterotypes.** Only showing genes with significant differential abundance between the enterotypes and at a relative abundance >0.1%. Data is expressed as relative abundance (%).

| Metagenome | Enterotype A | Enterotype B | Enterotype C |
| --- | --- | --- | --- |
| Toluene degradation | 0.106198551 | 0.060030432 | 0.094655493 |
| Biosynthesis of ansamycins | 0.107101939 | 0.133109478 | 0.11075408 |
| Taurine and hypotaurine metabolism | 0.107122443 | 0.095453446 | 0.103122182 |
| D-Alanine metabolism | 0.116317945 | 0.100727842 | 0.109559976 |
| Bacterial toxins | 0.117170407 | 0.114100265 | 0.106315648 |
| Aminobenzoate degradation | 0.117493381 | 0.096306109 | 0.101096077 |
| Metabolism of cofactors and vitamins | 0.119099672 | 0.096725994 | 0.106634601 |
| Tropane, piperidine and pyridine alkaloid biosynthesis | 0.119350412 | 0.110233854 | 0.116559547 |
| Phenylpropanoid biosynthesis | 0.122733997 | 0.173270859 | 0.132238771 |
| Tetracycline biosynthesis | 0.124633376 | 0.149703409 | 0.129817748 |
| RNA transport | 0.136184843 | 0.162419683 | 0.143037556 |
| Novobiocin biosynthesis | 0.140669352 | 0.136984808 | 0.139312338 |
| Lipid metabolism | 0.14201646 | 0.157565948 | 0.145191636 |
| Carbohydrate metabolism | 0.145078411 | 0.181877285 | 0.146915196 |
| Chloroalkane and chloroalkene degradation | 0.153937269 | 0.193578409 | 0.167685195 |
| Tuberculosis | 0.154663759 | 0.154256701 | 0.160992436 |
| D-Glutamine and D-glutamate metabolism | 0.157827828 | 0.142076844 | 0.156039807 |
| Plant-pathogen interaction | 0.158221561 | 0.180811625 | 0.161721237 |
| Peroxisome | 0.164731557 | 0.145309113 | 0.159791441 |
| Phenylalanine metabolism | 0.166766424 | 0.167483313 | 0.162562918 |
| RNA polymerase | 0.185995416 | 0.172052699 | 0.182643822 |
| Amino acid metabolism | 0.186063227 | 0.190667719 | 0.178072328 |
| Inorganic ion transport and metabolism | 0.188251273 | 0.172045524 | 0.170528043 |
| Sphingolipid metabolism | 0.192911876 | 0.213138679 | 0.199179492 |
| Benzoate degradation | 0.195279903 | 0.196291906 | 0.17171821 |
| Cell motility and secretion | 0.198908866 | 0.149123365 | 0.173815194 |
| Restriction enzyme | 0.201718469 | 0.177736025 | 0.198370461 |
| Vitamin B6 metabolism | 0.2023218 | 0.184029255 | 0.22534626 |
| Glutathione metabolism | 0.213145436 | 0.158804176 | 0.212601271 |
| Valine, leucine and isoleucine degradation | 0.223477778 | 0.186307936 | 0.189378747 |
| Ubiquinone and other terpenoid-quinone biosynthesis | 0.224885595 | 0.101191838 | 0.227167933 |
| Other transporters | 0.241551704 | 0.255683058 | 0.250736646 |
| Cyanoamino acid metabolism | 0.253410866 | 0.291316741 | 0.262455866 |
| Sulfur relay system | 0.255017425 | 0.274101563 | 0.239256312 |
| Sulfur metabolism | 0.263314899 | 0.261508996 | 0.243480632 |
| Protein kinases | 0.267827283 | 0.316825201 | 0.254884673 |
| Riboflavin metabolism | 0.269001111 | 0.231300367 | 0.279521976 |
| C5-Branched dibasic acid metabolism | 0.321052934 | 0.342875312 | 0.306375706 |
| Lipopolysaccharide biosynthesis | 0.331377636 | 0.137243952 | 0.31963243 |
| Prenyltransferases | 0.355676778 | 0.303687975 | 0.36777331 |
| Flagellar assembly | 0.356681079 | 0.425621791 | 0.301052606 |
| Cytoskeleton proteins | 0.363497564 | 0.419240628 | 0.389409929 |
| Glycerolipid metabolism | 0.36501286 | 0.418039143 | 0.35757371 |
| Pores ion channels | 0.370308689 | 0.231568333 | 0.347576244 |
| Glycosyltransferases | 0.382116926 | 0.303683903 | 0.374237027 |
| Phosphotransferase system (PTS) | 0.386193095 | 0.41750069 | 0.349710308 |
| Bacterial chemotaxis | 0.398524254 | 0.538365453 | 0.383298635 |
| Pentose and glucuronate interconversions | 0.40825608 | 0.501800481 | 0.423608927 |
| Photosynthesis | 0.429702332 | 0.450411764 | 0.43279851 |
| Photosynthesis proteins | 0.434133101 | 0.454471878 | 0.434615917 |
| Nucleotide excision repair | 0.434636726 | 0.405893606 | 0.444028472 |
| Lipopolysaccharide biosynthesis proteins | 0.435138876 | 0.214923407 | 0.40466339 |
| Folate biosynthesis | 0.439622045 | 0.364072464 | 0.431008502 |
| Signal transduction mechanisms | 0.446392698 | 0.489482084 | 0.448666742 |
| Nicotinate and nicotinamide metabolism | 0.460786064 | 0.43985221 | 0.48565871 |
| Glyoxylate and dicarboxylate metabolism | 0.482280964 | 0.498317987 | 0.454888948 |
| RNA degradation | 0.486080125 | 0.451643109 | 0.497038797 |
| Fatty acid biosynthesis | 0.487751413 | 0.489916511 | 0.487554215 |
| Thiamine metabolism | 0.508386211 | 0.535767228 | 0.518575005 |
| Glycerophospholipid metabolism | 0.550897849 | 0.599229643 | 0.556716778 |
| Cell cycle - Caulobacter | 0.551082252 | 0.527196867 | 0.568860285 |
| Membrane and intracellular structural molecules | 0.561686508 | 0.397458683 | 0.578049211 |
| Histidine metabolism | 0.563007349 | 0.618068712 | 0.569910827 |
| Translation factors | 0.590700662 | 0.542695569 | 0.601534576 |
| Sporulation | 0.603447131 | 0.988968496 | 0.710664031 |
| Terpenoid backbone biosynthesis | 0.627811939 | 0.590491174 | 0.638653698 |
| Citrate cycle (TCA cycle) | 0.645739656 | 0.510832123 | 0.658284721 |
| Protein export | 0.653469446 | 0.606938033 | 0.645428165 |
| Pantothenate and CoA biosynthesis | 0.657199323 | 0.667495963 | 0.643913959 |
| Carbon fixation in photosynthetic organisms | 0.662219753 | 0.660565199 | 0.684445582 |
| One carbon pool by folate | 0.688978587 | 0.622333388 | 0.717281811 |
| Galactose metabolism | 0.695737848 | 0.756519675 | 0.702807356 |
| Protein folding and associated processing | 0.697277133 | 0.594921729 | 0.641674727 |
| DNA replication | 0.713553529 | 0.653963866 | 0.732340684 |
| Valine, leucine and isoleucine biosynthesis | 0.744199705 | 0.801423591 | 0.711379214 |
| Replication, recombination and repair proteins | 0.79517285 | 0.817880534 | 0.82223026 |
| Lysine biosynthesis | 0.820656272 | 0.89050111 | 0.849939289 |
| Pentose phosphate pathway | 0.822503789 | 0.920088006 | 0.848408184 |
| Others | 0.832024944 | 0.865156591 | 0.790318896 |
| Bacterial motility proteins | 0.847288999 | 1.035827189 | 0.744485831 |
| Fructose and mannose metabolism | 0.864171674 | 0.96673157 | 0.932733641 |
| Energy metabolism | 0.869769035 | 0.858782699 | 0.912636494 |
| Mismatch repair | 0.885733849 | 0.839740232 | 0.917421607 |
| Phenylalanine, tyrosine and tryptophan biosynthesis | 0.887626126 | 0.916874933 | 0.87957795 |
| Peptidoglycan biosynthesis | 0.90733998 | 0.852041372 | 0.945288472 |
| Starch and sucrose metabolism | 0.94084789 | 1.076221537 | 0.99878264 |
| Porphyrin and chlorophyll metabolism | 0.949333788 | 1.072975501 | 0.86031707 |
| Transcription machinery | 0.963868807 | 1.041025383 | 1.041290983 |
| Translation proteins | 0.974177026 | 0.92563638 | 0.9967019 |
| Pyruvate metabolism | 0.989145312 | 1.0153334 | 0.963834169 |
| Homologous recombination | 1.022016924 | 0.945226122 | 1.056921313 |
| Carbon fixation pathways in prokaryotes | 1.026368357 | 0.922784149 | 1.05213981 |
| Alanine, aspartate and glutamate metabolism | 1.044107382 | 1.055865222 | 1.079197972 |
| Chaperones and folding catalysts | 1.081464173 | 0.977258161 | 1.128781194 |
| Glycolysis / Gluconeogenesis | 1.089810159 | 1.116464452 | 1.102517953 |
| Arginine and proline metabolism | 1.106663486 | 1.230986637 | 1.113711492 |
| Oxidative phosphorylation | 1.157146407 | 1.100004698 | 1.216396262 |
| Methane metabolism | 1.213124302 | 1.321463901 | 1.222654563 |
| Two-component system | 1.267457311 | 1.431909297 | 1.174567991 |
| Aminoacyl-tRNA biosynthesis | 1.306263328 | 1.232433596 | 1.297755819 |
| DNA replication proteins | 1.306968778 | 1.231399832 | 1.370737448 |
| Function unknown | 1.317343334 | 1.150498675 | 1.194898938 |
| Amino sugar and nucleotide sugar metabolism | 1.419524683 | 1.416178597 | 1.476290989 |
| Ribosome Biogenesis | 1.518080955 | 1.475399314 | 1.561111447 |
| Transcription factors | 1.522973402 | 1.820291159 | 1.568336943 |
| Amino acid related enzymes | 1.576831196 | 1.502336991 | 1.585340214 |
| Chromosome | 1.665807749 | 1.615463018 | 1.77492405 |
| Peptidases | 1.938788002 | 1.889063551 | 2.018633232 |
| Pyrimidine metabolism | 1.994873511 | 1.843116079 | 2.034097195 |
| Purine metabolism | 2.400988583 | 2.184718843 | 2.406248067 |
| Ribosome | 2.641623766 | 2.425760032 | 2.706642095 |
| DNA repair and recombination proteins | 3.044712937 | 2.857649458 | 3.109353704 |
| ABC transporters | 3.172686477 | 3.594643765 | 2.976403852 |
| General function prediction only | 3.576947869 | 3.606373296 | 3.639452521 |
| Transporters | 6.243499168 | 7.371279796 | 5.993362952 |

**Supplementary Table 3: Relative abundances of predicted metagenome categorized by function across fecal enterotypes.** Only showing genes with significant differential abundance between the enterotypes and at a relative abundance >0.1%. Data is expressed as relative abundance (%).

| Metagenome | Enterotype A | Enterotype B | Enterotype C |
| --- | --- | --- | --- |
| Toluene degradation | 0.106198551 | 0.060030432 | 0.094655493 |
| Biosynthesis of ansamycins | 0.107101939 | 0.133109478 | 0.11075408 |
| Taurine and hypotaurine metabolism | 0.107122443 | 0.095453446 | 0.103122182 |
| D-Alanine metabolism | 0.116317945 | 0.100727842 | 0.109559976 |
| Bacterial toxins | 0.117170407 | 0.114100265 | 0.106315648 |
| Aminobenzoate degradation | 0.117493381 | 0.096306109 | 0.101096077 |
| Metabolism of cofactors and vitamins | 0.119099672 | 0.096725994 | 0.106634601 |
| Tropane, piperidine and pyridine alkaloid biosynthesis | 0.119350412 | 0.110233854 | 0.116559547 |
| Phenylpropanoid biosynthesis | 0.122733997 | 0.173270859 | 0.132238771 |
| Tetracycline biosynthesis | 0.124633376 | 0.149703409 | 0.129817748 |
| RNA transport | 0.136184843 | 0.162419683 | 0.143037556 |
| Novobiocin biosynthesis | 0.140669352 | 0.136984808 | 0.139312338 |
| Lipid metabolism | 0.14201646 | 0.157565948 | 0.145191636 |
| Carbohydrate metabolism | 0.145078411 | 0.181877285 | 0.146915196 |
| Chloroalkane and chloroalkene degradation | 0.153937269 | 0.193578409 | 0.167685195 |
| Tuberculosis | 0.154663759 | 0.154256701 | 0.160992436 |
| D-Glutamine and D-glutamate metabolism | 0.157827828 | 0.142076844 | 0.156039807 |
| Plant-pathogen interaction | 0.158221561 | 0.180811625 | 0.161721237 |
| Peroxisome | 0.164731557 | 0.145309113 | 0.159791441 |
| Phenylalanine metabolism | 0.166766424 | 0.167483313 | 0.162562918 |
| RNA polymerase | 0.185995416 | 0.172052699 | 0.182643822 |
| Amino acid metabolism | 0.186063227 | 0.190667719 | 0.178072328 |
| Inorganic ion transport and metabolism | 0.188251273 | 0.172045524 | 0.170528043 |
| Sphingolipid metabolism | 0.192911876 | 0.213138679 | 0.199179492 |
| Benzoate degradation | 0.195279903 | 0.196291906 | 0.17171821 |
| Cell motility and secretion | 0.198908866 | 0.149123365 | 0.173815194 |
| Restriction enzyme | 0.201718469 | 0.177736025 | 0.198370461 |
| Vitamin B6 metabolism | 0.2023218 | 0.184029255 | 0.22534626 |
| Glutathione metabolism | 0.213145436 | 0.158804176 | 0.212601271 |
| Valine, leucine and isoleucine degradation | 0.223477778 | 0.186307936 | 0.189378747 |
| Ubiquinone and other terpenoid-quinone biosynthesis | 0.224885595 | 0.101191838 | 0.227167933 |
| Other transporters | 0.241551704 | 0.255683058 | 0.250736646 |
| Cyanoamino acid metabolism | 0.253410866 | 0.291316741 | 0.262455866 |
| Sulfur relay system | 0.255017425 | 0.274101563 | 0.239256312 |
| Sulfur metabolism | 0.263314899 | 0.261508996 | 0.243480632 |
| Protein kinases | 0.267827283 | 0.316825201 | 0.254884673 |
| Riboflavin metabolism | 0.269001111 | 0.231300367 | 0.279521976 |
| C5-Branched dibasic acid metabolism | 0.321052934 | 0.342875312 | 0.306375706 |
| Lipopolysaccharide biosynthesis | 0.331377636 | 0.137243952 | 0.31963243 |
| Prenyltransferases | 0.355676778 | 0.303687975 | 0.36777331 |
| Flagellar assembly | 0.356681079 | 0.425621791 | 0.301052606 |
| Cytoskeleton proteins | 0.363497564 | 0.419240628 | 0.389409929 |
| Glycerolipid metabolism | 0.36501286 | 0.418039143 | 0.35757371 |
| Pores ion channels | 0.370308689 | 0.231568333 | 0.347576244 |
| Glycosyltransferases | 0.382116926 | 0.303683903 | 0.374237027 |
| Phosphotransferase system (PTS) | 0.386193095 | 0.41750069 | 0.349710308 |
| Bacterial chemotaxis | 0.398524254 | 0.538365453 | 0.383298635 |
| Pentose and glucuronate interconversions | 0.40825608 | 0.501800481 | 0.423608927 |
| Photosynthesis | 0.429702332 | 0.450411764 | 0.43279851 |
| Photosynthesis proteins | 0.434133101 | 0.454471878 | 0.434615917 |
| Nucleotide excision repair | 0.434636726 | 0.405893606 | 0.444028472 |
| Lipopolysaccharide biosynthesis proteins | 0.435138876 | 0.214923407 | 0.40466339 |
| Folate biosynthesis | 0.439622045 | 0.364072464 | 0.431008502 |
| Signal transduction mechanisms | 0.446392698 | 0.489482084 | 0.448666742 |
| Nicotinate and nicotinamide metabolism | 0.460786064 | 0.43985221 | 0.48565871 |
| Glyoxylate and dicarboxylate metabolism | 0.482280964 | 0.498317987 | 0.454888948 |
| RNA degradation | 0.486080125 | 0.451643109 | 0.497038797 |
| Fatty acid biosynthesis | 0.487751413 | 0.489916511 | 0.487554215 |
| Thiamine metabolism | 0.508386211 | 0.535767228 | 0.518575005 |
| Glycerophospholipid metabolism | 0.550897849 | 0.599229643 | 0.556716778 |
| Cell cycle - Caulobacter | 0.551082252 | 0.527196867 | 0.568860285 |
| Membrane and intracellular structural molecules | 0.561686508 | 0.397458683 | 0.578049211 |
| Histidine metabolism | 0.563007349 | 0.618068712 | 0.569910827 |
| Translation factors | 0.590700662 | 0.542695569 | 0.601534576 |
| Sporulation | 0.603447131 | 0.988968496 | 0.710664031 |
| Terpenoid backbone biosynthesis | 0.627811939 | 0.590491174 | 0.638653698 |
| Citrate cycle (TCA cycle) | 0.645739656 | 0.510832123 | 0.658284721 |
| Protein export | 0.653469446 | 0.606938033 | 0.645428165 |
| Pantothenate and CoA biosynthesis | 0.657199323 | 0.667495963 | 0.643913959 |
| Carbon fixation in photosynthetic organisms | 0.662219753 | 0.660565199 | 0.684445582 |
| One carbon pool by folate | 0.688978587 | 0.622333388 | 0.717281811 |
| Galactose metabolism | 0.695737848 | 0.756519675 | 0.702807356 |
| Protein folding and associated processing | 0.697277133 | 0.594921729 | 0.641674727 |
| DNA replication | 0.713553529 | 0.653963866 | 0.732340684 |
| Valine, leucine and isoleucine biosynthesis | 0.744199705 | 0.801423591 | 0.711379214 |
| Replication, recombination and repair proteins | 0.79517285 | 0.817880534 | 0.82223026 |
| Lysine biosynthesis | 0.820656272 | 0.89050111 | 0.849939289 |
| Pentose phosphate pathway | 0.822503789 | 0.920088006 | 0.848408184 |
| Others | 0.832024944 | 0.865156591 | 0.790318896 |
| Bacterial motility proteins | 0.847288999 | 1.035827189 | 0.744485831 |
| Fructose and mannose metabolism | 0.864171674 | 0.96673157 | 0.932733641 |
| Energy metabolism | 0.869769035 | 0.858782699 | 0.912636494 |
| Mismatch repair | 0.885733849 | 0.839740232 | 0.917421607 |
| Phenylalanine, tyrosine and tryptophan biosynthesis | 0.887626126 | 0.916874933 | 0.87957795 |
| Peptidoglycan biosynthesis | 0.90733998 | 0.852041372 | 0.945288472 |
| Starch and sucrose metabolism | 0.94084789 | 1.076221537 | 0.99878264 |
| Porphyrin and chlorophyll metabolism | 0.949333788 | 1.072975501 | 0.86031707 |
| Transcription machinery | 0.963868807 | 1.041025383 | 1.041290983 |
| Translation proteins | 0.974177026 | 0.92563638 | 0.9967019 |
| Pyruvate metabolism | 0.989145312 | 1.0153334 | 0.963834169 |
| Homologous recombination | 1.022016924 | 0.945226122 | 1.056921313 |
| Carbon fixation pathways in prokaryotes | 1.026368357 | 0.922784149 | 1.05213981 |
| Alanine, aspartate and glutamate metabolism | 1.044107382 | 1.055865222 | 1.079197972 |
| Chaperones and folding catalysts | 1.081464173 | 0.977258161 | 1.128781194 |
| Glycolysis / Gluconeogenesis | 1.089810159 | 1.116464452 | 1.102517953 |
| Arginine and proline metabolism | 1.106663486 | 1.230986637 | 1.113711492 |
| Oxidative phosphorylation | 1.157146407 | 1.100004698 | 1.216396262 |
| Methane metabolism | 1.213124302 | 1.321463901 | 1.222654563 |
| Two-component system | 1.267457311 | 1.431909297 | 1.174567991 |
| Aminoacyl-tRNA biosynthesis | 1.306263328 | 1.232433596 | 1.297755819 |
| DNA replication proteins | 1.306968778 | 1.231399832 | 1.370737448 |
| Function unknown | 1.317343334 | 1.150498675 | 1.194898938 |
| Amino sugar and nucleotide sugar metabolism | 1.419524683 | 1.416178597 | 1.476290989 |
| Ribosome Biogenesis | 1.518080955 | 1.475399314 | 1.561111447 |
| Transcription factors | 1.522973402 | 1.820291159 | 1.568336943 |
| Amino acid related enzymes | 1.576831196 | 1.502336991 | 1.585340214 |
| Chromosome | 1.665807749 | 1.615463018 | 1.77492405 |
| Peptidases | 1.938788002 | 1.889063551 | 2.018633232 |
| Pyrimidine metabolism | 1.994873511 | 1.843116079 | 2.034097195 |
| Purine metabolism | 2.400988583 | 2.184718843 | 2.406248067 |
| Ribosome | 2.641623766 | 2.425760032 | 2.706642095 |
| DNA repair and recombination proteins | 3.044712937 | 2.857649458 | 3.109353704 |
| ABC transporters | 3.172686477 | 3.594643765 | 2.976403852 |
| General function prediction only | 3.576947869 | 3.606373296 | 3.639452521 |
| Transporters | 6.243499168 | 7.371279796 | 5.993362952 |

**Supplementary Table 4: Vaginal pH.**

| Animal_ID | Vaginal pH |
| --- | --- |
| VSAA1003 | 7.5 |
| VSAA1006 | 7 |
| VSAA1009 | 7 |
| VSAA1010 | 7.5 |
| VSAA2001 | 7.5 |
| VSAA2003 | 6 |
| VSAA2005 | 7.5 |
| VSAA2015 | 8 |
| VSAA2021 | 8.5 |
| VSAA4005 | 7 |
| VSAB1006 | 5.5 |
| VSAB1008 | 6.5 |
| VSAB2009 | 7.5 |
| VSAB2011 | 6.5 |
| VSAB2012 | 6 |
| VSAB2013 | 6.5 |
| VSAB2021 | 6.5 |
| VSAB2023 | 5 |
| VSAB3004 | 6.5 |
| VSAC1004 | 7.5 |
| VSAC1015 | 7 |
| VSAC2002 | 5.5 |
| VSAC2006 | 7.5 |
| VSAC3003 | 7.5 |
| VSAC4004 | 7.5 |
| VSAE1001 | 6 |
| VSAE1004 | 7 |
| VSAE1006 | 7 |
| VSAE2001 | 7.5 |
| VSAE2005 | 7.5 |
| VSAE2006 | 7 |
| VSAE2007 | 8 |
| VSAE2009 | 7 |
| VSAE2011 | 5.5 |
| VSAE3001 | 7.5 |
| VSAE3003 | 6.5 |
| VSAF1004 | 6 |
| VSAF1006 | 7.5 |
| VSAF1009 | 8 |
| VSAF1011 | 6.5 |
| VSAF1015 | 8 |
| VSAG1003 | 7.5 |
| VSAG1010 | 7 |
| VSAG2003 | 7.5 |
| VSAG2005 | 6.5 |
| VSAH1003 | 7.5 |
