# Supplementary material for: Shifts in microbial diversity, composition, and functionality in the gut and genital microbiome during a natural SIV infection in vervet monkeys
Source: Microbiome. 2020 Nov 6;8:154. doi: 10.1186/s40168-020-00928-4 (PMC7648414; doi:10.1186/s40168-020-00928-4)
Supplement: Supplementary file 4 — Additional file 3 Additional Data 1. [file 40168_2020_928_MOESM3_ESM.docx]

**Additional Data 1**

**A) Natural composition of the microbiome**

The gut microbiome samples in the rectal and fecal sample sets each formed a tight cluster, which, as expected, partially overlapped. In contrast to the gut microbiome, the genital microbiome from penile and vaginal samples were grouped and jointly formed a less compact cluster. Given that the genital samples came almost exclusively from adult individuals, our results were concordant with the expected exchange in microbes between males and females through heterosexual contact. Some genital samples appeared to be intermediate between gut and genital clusters, thus suggesting the presence of fecal bacteria in the genital area, which probably represented residual populations rather than temporary contamination, because we applied very stringent cleaning procedures for the rectal, perineal and vaginal areas. Previously, intermixing of vaginal samples with gut samples was shown to be more common in macaques than humans [[1]](https://paperpile.com/c/WGij8J/Kn3u), thus suggesting a role of inter-specific differences in hygiene and sanitary conditions and lifestyle.

Firmicutes and Bacteroidetes were the most predominant phyla in microbial communities across all four body sites studied (Figure 1C). Firmicutes were most abundant in the penile microbiome (62.2%), followed by fecal (49.2%), vaginal (45.8%) and rectal (41%) microbiomes. Bacteroidetes constituted 33.8% of the fecal, 33% of the rectal, 24.9% of the vaginal and 19% of the penile microbiomes. Phyla with lower relative abundance were differentially distributed between the gut and genital microbiomes. Fusobacteria and Actinobacteria were enriched in the genital microbiome (21.9% and 34.9%, respectively, in the vaginal microbiome, and 9.3% and 42%, respectively, in the penile microbiome), whereas Spirochaetes and Proteobacteria were overrepresented in gut microbiome (3.6% and 7.3%, respectively, in the fecal microbiome, and 6% and 5.7%, respectively, in the rectal microbiome). The least abundant phyla, Epsilonbacteraeota and Cyanobacteria, were enriched in the rectal microbiome compared with the microbiomes from other body sites.

**B) Vaginal microbiota composition, correlates and comparison to other primates**

In the vaginal microbiome, Firmicutes were the most abundant phylum in the African vervets studied here (Figure 1C), as previously observed in humans and several NHP species, with the exception of NHPs living in captive settings (vervets and chimpanzees), in which Fusobacteria exceeds Firmicutes (Supplementary Figure 3A) [[1,2]](https://paperpile.com/c/WGij8J/EmkJ+Kn3u). The Caribbean vervets have been found to have the lowest diversity in the vaginal microbiome among NHPs, yet their diversity is still higher than that in humans. We were unable to perform a formal comparison of microbial diversity measures between previous studies and our study, because of technical differences (we evaluated a different 16S rRNA gene region), but we inspected the vaginal microbiome composition at the genus level in South African vervets (Supplementary Figure 3B) and Caribbean-origin vervets, both from free-ranging Caribbean populations and from a US-based research colony (Supplementary Figure 3C), and observed a striking difference in the vaginal microbiome composition between these populations.

We observed two vagitypes (A and B) in the African vervets. They may have been associated with individual differences, the stage of the uterine cycle or menstrual cycle, or environmental factors. We do not have data from the studied individuals on the cycle stage to relate to the vagitypes. These phases are not readily observable in the field setting because vervets have attenuated menstruation with very light menstrual flow[[3]](https://paperpile.com/c/WGij8J/vXm67), among other reasons. However, we measured vaginal pH, which in mammals varies along the estrogen production cycles of the ovaries with lowest vaginal pH observed during periods of high estrogen levels [[4]](https://paperpile.com/c/WGij8J/FpWFC). The vaginal pH in vervets ranged from 5 to 8.5 with an average pH of 6.98 + 1.27 (Supplementary Table 4)). The near neutral vaginal pH in vervets is in contrast to the moderately acidic vaginal pH seen in most humans (average pH of 3.8-4.5) and is most likely the result of near absence of *Lactobacillus* in vervets. In humans, highly abundant *Lactobacillus* produces lactic acid which lowers vaginal pH and creates an acidic environment that has antimicrobial properties [[4,5]](https://paperpile.com/c/WGij8J/FpWFC+8MLfn), while African vervets showed an extremely low abundance of *Lactobacillus* in the vaginal microbiome (0.06 %), concordant with the low levels of *Lactobacillus* previously noted in Caribbean vervets and several other NHPs [[2]](https://paperpile.com/c/WGij8J/EmkJ). The vaginal pH did not have an effect on the overall bacterial diversity in the vervet vagina. However, acidic environment was associated with an increase in *Aerococcus*, *Trichococcus*, and *Streptoccocus* (characteristic to vagitype A), and a decrease in *Porphyromonas* (Supplementary Figure 4). In accordance with this observation, vagitype A was associated with more acidic pH (mean pH = 6.22+Stdev 0.712), while vagitype B was associated with more alkaline pH (mean pH = 7.16 + Stdev 0.68) (p-value 0.002). Given that lowered pH may result from increased estrogen levels [[4]](https://paperpile.com/c/WGij8J/FpWFC), the vervet vagitypes could be potentially shaped by estrogen differences due to cycle and age-related ovarian activity, although direct assessment of hormone levels would be needed to establish such a link and differentiate the effects of estrogens from other causes of vaginal dysbiosis.

In the vaginal microbiota in the Caribbean-origin vervets [[2]](https://paperpile.com/c/WGij8J/EmkJ), one community structure was dominated by anaerobic *Sneathia* from the phylum Fusobacteria, and the other was dominated by facultative anaerobic *Aerococcus* from the phylum Firmicutes (Supplementary Figure 3C). In the *Aerococcus* dominated ecosystem, the relative abundance of this microbe ranged between 62.8% and 91.2%, and in the *Sneathia-dominated* ecosystem, the relative abundance of this bacterium ranged from 59.9% to 77.7%. In our South African vervets, Sneathia was not detectable, whereas Aerococcus accounted for only 6.54% of the total microbiota in SIV infected and uninfected monkeys (Supplementary Figure 3B). Aerococcus, however, was seen in higher prevalence in vervet monkeys of vaginotype A (13.7%-37.7%) but still lower than Caribbean-origin vervets. Taken together, the vaginal community composition at the individual level was characterized by a dichotomic pattern in most of the Caribbean-origin vervets and our South African vervets yet with remarkably different composition and community structures between these two geographic groups.

Several possible factors may have contributed to this dramatic shift in the vaginal microbiome composition. One hypothesis is that the shift may have resulted from the population bottleneck associated with establishing the Caribbean vervet populations from the west African vervets, followed by nearly 300 years of isolation. A second hypothesis is that these differences arose even earlier during speciation and radiation of the genus *Chlorocebus*, which led to the emergence of the west African vervet species *Ch. sabaeus* (the ancestral population for the Caribbean vervets) and the South African vervet species *Ch. pygerythrus*. A third hypothesis is that these differences may result from the distinct environmental conditions in which African vervets and Caribbean-origin vervets live, yet this possibility seems rather unlikely because Caribbean-origin vervets from different locations (natural populations and a US-based colony) share similar microbial profiles, which are distinct from those observed in Africa. The extent to which environmental or host genetic factors confer these differences requires further investigation. These hypotheses could be tested in the future by assessing the microbiome in West African vervets. We did not observe an analogous effect in the rectal microbiome in our South African vervets and Caribbean vervets characterized in Amato et al. 2015 (Supplementary Figure 16). Why the vaginal microbiome within the genus *Chlorocebus* is more prone to shifts in composition than the rectal microbiome remains unclear. One possible explanation is that, given that vaginal microbiome diversity is strongly correlated with host promiscuous behavior both in mouse and primates [[2,6]](https://paperpile.com/c/WGij8J/EmkJ+4N1xw), composition may shift with novel introductions based on copulatory patterns. On a related note, the severe bottleneck in the Caribbean vervets [[7]](https://paperpile.com/c/WGij8J/K8XE8) which reduced the number of mating partners, could have contributed to the reduced overall diversity of the vaginal microbiome we observed. The lower diversity of the vaginal microbiome relative to the gut may also make it more susceptible to such demographic effects.

**C) Geography-related variation in microbiota**

We assessed the associations between the microbiome and two provinces, KZN and FS. Differential abundance testing between the KZN and FS provinces showed association of several “moderately” abundant microbial taxa with geography (Supplementary Figure 5E). In the gut, *Helicobacter* was underrepresented and *Treponema* was overrepresented in the rectum of KZN relative to FS, whereas *Alloprevotella* was overrepresented in feces in KZN relative to FS. Several genera from the phylum Firmicutes were differentially abundant between the provinces in the genital ecosystems. *Finegoldia* and *Peptoniphilus* were overrepresented in the penile microbiome, and *Aerococcus*, *Streptococcus*, *Peptoniphilus*, *Trichococcus* and *Anaerococcus* were underrepresented in the vaginal microbiome in KZN compared with FS. The regional differences in abundances of individual genera reflect more complex differences in geographic distribution of vagitypes. Aerococcus, Trichococcus, and Anaerococcus were highly abundant in vaginotype A which was more common in FS (34.5%) and in KZN (5%). The extent to which these compositional shifts in microbial communities are driven by environmental or host genetic factors remains to be determined.

**D) Bioclimate-related variation in microbiota**

Previous studies had shown a link between genetic variation in genes involved in innate immunity and climatic variables (e.g. high rainfall) in South African vervets suggesting the role of climatic conditions in regulating host-associated microbial ecosystems [12]. We analyzed a correlation between vervet microbiome composition and major bioclimatic variables from the WorldClim data [35]. Two climatic variables, Mean Temp and Min Temp, were associated with significant differences in the microbiome composition while adjusting for sex, age, province, and SIV status. Clustering of vaginal and rectal samples was associated with both Min and Mean Temps, and fecal sample clustering was associated with Min Temp (Supplementary Figure 8). Overall, Min Temp had the greatest changes, and vaginal samples were most affected by temperature. Several of the bacterial taxa associated with vagitype A (Streptococcus, Trichococcus, and Aerococcus) were enriched in the lowest tertile of annual Mean Temp p-value < 0.001, and the mid tertile of Min Temp for the coldest month (p-value < 0.0001), which comprised 90% of all samples with vagitype A (Supplementary Figure 9). The associations between vaginal microbiome and both biome and temperature are not surprising, given that these environmental factors are related. Min Temp was associated with abundances of Helicobacter (decrease) and Anaerovibrio (increase) in the rectal microbiome.

Alpha diversity was significantly increased in the penile microbiome of the monkeys living at the lowest and mid tertiles compared to highest tertial of Min and Mean Temps (p-value < 0.05); however, this analysis was based on a relatively small number of males living under these conditions and thus these results should be interpreted with caution.

Taken together, we observed that environmental temperature influences mostly the microbiomes associated with the local mucosa of outer body orifices with more exposure to environmental factors, while there is no such observable effect on the microbiome of fecal samples, which seem to represent a more “internal” microbiome. The sensitivity of host microbiome to environmental temperatures may possibly result from the direct effect of temperature on the host-associated microbiome or effects on the host (for example, its immune functions or metabolism) or on available food resources (and therefore different energy and nutrient sources for host-associated microbiota). Given the lack of apparent effects of temperature on the fecal microbiome, diet is probably not the main factor for mediating the effects of temperature on rectal and vaginal microbiomes.

**References**

[1. Chen Z, Yeoh YK, Hui M, Wong PY, Chan MCW, Ip M, et al. Diversity of macaque microbiota compared to the human counterparts. Sci Rep. 2018;8:15573.](http://paperpile.com/b/WGij8J/Kn3u)

[2. Yildirim S, Yeoman CJ, Janga SC, Thomas SM, Ho M, Leigh SR, et al. Primate vaginal microbiomes exhibit species specificity without universal Lactobacillus dominance. ISME J. 2014;8:2431–44.](http://paperpile.com/b/WGij8J/EmkJ)

[3. Carroll RL, Mah K, Fanton JW, Maginnis GN, Brenner RM, Slayden OD. Assessment of menstruation in the vervet (Cercopithecus aethiops). Am J Primatol. 2007;69:901–16.](http://paperpile.com/b/WGij8J/vXm67)

[4. Miller EA, Beasley DE, Dunn RR, Archie EA. Lactobacilli Dominance and Vaginal pH: Why Is the Human Vaginal Microbiome Unique? Front Microbiol. 2016;7:1936.](http://paperpile.com/b/WGij8J/FpWFC)

[5. O’Hanlon DE, Come RA, Moench TR. Vaginal pH measured in vivo: lactobacilli determine pH and lactic acid concentration. BMC Microbiol. 2019;19:13.](http://paperpile.com/b/WGij8J/8MLfn)

[6. Glavan TW, Gaulke CA, Santos Rocha C, Sankaran-Walters S, Hirao LA, Raffatellu M, et al. Gut immune dysfunction through impaired innate pattern recognition receptor expression and gut microbiota dysbiosis in chronic SIV infection. Mucosal Immunol. 2016;9:677–88.](http://paperpile.com/b/WGij8J/4N1xw)

[7. Warren WC, Jasinska AJ, García-Pérez R, Svardal H, Tomlinson C, Rocchi M, et al. The genome of the vervet (Chlorocebus aethiops sabaeus). Genome Res. 2015;25:1921–33.](http://paperpile.com/b/WGij8J/K8XE8)
